# VSTM2A suppresses colorectal cancer and antagonizes Wnt signaling receptor LRP6

**DOI:** 10.7150/thno.34989

**Published:** 2019-08-21

**Authors:** Yujuan Dong, Yanquan Zhang, Wei Kang, Guoping Wang, Huarong Chen, Akira Higashimori, Geicho Nakatsu, Minnie Go, Joanna HM Tong, Shu Zheng, Ka Fai To, Joseph JY Sung, Xiaoyong Yang, Simon SM Ng, Jun Yu

**Affiliations:** 1 Institute of Digestive Disease and Department of Medicine and Therapeutics, State Key Laboratory of Digestive Disease and Li Ka Shing Institute of Health Sciences, CUHK-Shenzhen Research Institute, The Chinese University of Hong Kong, Hong Kong; 2 Department of Surgery, The Chinese University of Hong Kong, Hong Kong; 3 Department of Anatomical and Cellular Pathology, The Chinese University of Hong Kong, Hong Kong; 4 Department of Gastroenterology, Osaka City University Graduate School of Medicine, Osaka, Japan; 5 Department of Gastroenterology, Zhejiang University, China; 6 Program in Integrative Cell Signaling and Neurobiology of Metabolism, Department of Comparative Medicine, Yale University School of Medicine, New Haven, Connecticut 06520, USA

**Keywords:** VSTM2A, LRP6, Wnt signaling, colorectal cancer prognosis.

## Abstract

Hyperactivation of Wnt/β-catenin signaling pathway is a critical step in colorectal tumorigenesis. In this study, we identified that V-set and transmembrane domain containing 2A (VSTM2A) was a top-downregulated secreted protein that negatively regulated Wnt singling pathways in colorectal cancer (CRC). We investigated the functional mechanisms and clinical implication of VSTM2A in CRC.

**Methods:** Function of VSTM2A was investigated *in vitro* and *in vivo*. VSTM2A binding partner was identified by mass spectrometry, immunoprecipitation and Western blot. The clinical impact of VSTM2A was assessed in 355 CRC patients and TCGA cohort.

**Results:** VSTM2A protein was prominently silenced in CRC tumor tissues and cell lines mediated by its promoter hypermethylation. VSTM2A DNA promoter hypermethylation and VSTM2A protein downregulation was associated with poor survival of CRC patients. Ectopic expression of VSTM2A inhibited colon cancer cell lines and organoid growth, induced CRC cells apoptosis, inhibited cell migration and invasion, and suppressed growth of xenograft tumors in nude mice. VSTM2A was released from CRC cells through a canonical secretion pathway. Secreted VSTM2A significantly suppressed Wnt signaling pathway in colon cancer cells. Wnt signaling co-receptor LDL receptor related protein 6 (LRP6) was identified as a cell membrane binding partner of VSTM2A. Using deletion/mutation and immunoprecipitation, we demonstrated that VSTM2A bound to LRP6 E1-4 domain with its IgV domain. VSTM2A suppressed LRP6 phosphorylation in a time and dose dependent manner, and induced LRP6 endocytosis and lysosome-mediated degradation, which collectively contributing to the inactivation of Wnt signaling.

**Conclusions:** VSTM2A is a novel antagonist of canonical Wnt signaling by directly binding to LRP6 and induces LRP6 endocytosis and degradation. VSTM2A is a potential prognostic biomarker for the outcome of CRC patients.

## INTRODUCTION

Colorectal cancer (CRC) is the third most common cancer and ranks the second in terms of cancer mortality worldwide based on the newly released Global cancer statistics 2018 1. Both the incidence and death rate of CRC are increasing rapidly and maintaining an upward trend in Asian countries undergoing major development transition 2. It is important to identify new critical genes in the pathogenesis of CRC for developing effective treatments. Hyperactivation of Wnt/β-catenin signaling pathway is believed to be a critical step in colorectal tumorigenesis. Accumulated β-catenin enters into nucleus of the colon cancer cells and interacts with T-cell factor family transcription factors leading to activation of the Wnt pathway downstream target genes and promotes tumorigenesis 3. Wnt signaling is modulated mainly by Wnt agonists (Wnt family), and antagonists such as Dickkopfs (DKKs) and secreted frizzled-related proteins (SFRPs) 4, 5. However, other secreted proteins responsible for modulation of Wnt signaling are still largely unknown.

We analyzed 4642 predicted secreted proteins from the The Cancer Genome Atlas (TCGA) dataset for colon & rectum adenocarcinoma (COADREAD) gene expression by RNA sequencing and identified VSTM2A was one of the top-downregulated secreted proteins in CRC, but is readily expressed in normal colon tissue. The *VSTM2A* gene spans 27 kb of genomic sequence on chromosome 7 and consists of five exons. VSTM2A protein composes of 240 amino acid (aa), containing a potential 25-aa N-terminal signal peptide, two putative glycosylation sites, and a single immunoglobulin V-set domain. Previous study suggested a role of VSTM2A in the regulation of the preadipocyte cell differentiation 6. VSTM2A was markedly reduced in colorectal adenoma compared with the normal mucosa in a transcriptome profiling of 32 patients 7. However the role of VSTM2A in CRC tumorigenesis and its signaling pathway is unknown. In this study, we provide a series of experimental evidences that VSTM2A is a critical tumor suppressor in CRC and a novel antagonist of Wnt signaling by direct binding to Wnt co-receptor LRP6, suppressing LRP6 activation and inducing its endocytosis and degradation. We also revealed the clinical impact of VSTM2A in large scale cohorts of CRC patients.

## MATERIAL AND METHODS

### Colorectal tissue samples

Three cohorts of 355 CRC patients were included in this study. Forty-six paired primary CRC and adjacent non-tumor fresh specimens and 158 CRC paraffin blocks were obtained during operation of CRC patients diagnosed in the Prince of Wales Hospital, the Chinese University of Hong Kong from 2002 to 2017. A cohort of 151 primary colorectal tumors and adjacent normal tissue RNA were obtained from Zhejiang University, China. Colorectal cancer patients with age >18 were enrolled in this study. Pregnant or nursing patients were excluded. Written informed consents were obtained from subjects or their authorized representatives. The study protocol was approved by the Ethics Committee of the Chinese University of Hong Kong and Ethics Committee of Zhejiang University. This study was carried out in accordance with the Declaration of Helsinki of the World Medical Association.

### Cell lines and CRC patient-derived organoid model

The human CRC cell lines (colo205, DLD1, HCT116, HT29, LoVo, SW620, and SW1116), L cell and L Wnt-3A cell were purchased from the American Type Culture Collection (ATCC; Manassas, VA). 293T cell line was purchased from Invitrogen (Thermo Fisher Scientific, Waltham, MA). Cell lines were maintained according to the protocols from American Type Culture Collection. All cell lines were obtained between 2013 and 2015. Routine Mycoplasma testing was performed by PCR. Cells were grown for no more than 25 passages in total for any experiment. CRC patient-derived organoid model were kindly provided by Prof. Catherine O'Brien, Department of Surgery at University Health Network and cultured as described previously 8.

### Candidate extracellular genes screening and analysis of VSTM2A signatures in TCGA

A list of extracellular genes of 4,642 unique human proteins encoded by 3,641 unique genes were screened and evaluated in this study 9. To identify the dysregulated secreted protein in CRC, we analysed the publicly available datasets of TCGA-COADREAD gene expression by RNA sequencing on the Illumina HiSeq 2000 platform. To investigate the epigenetic regulation of VSTM2A gene expression in colorectal cancer, we analyzed TCGA datasets of methylome profiling on the Infinium DNA Methylation Array. IDAT files and raw mRNA sequence counts were obtained from the TCGA Data Portal (http://tcga-data.nci.nih.gov/tcga/).

### Cloning of VSTM2A and LRP6 expression vectors

Sequence corresponding to the open reading frame (ORF) of human *VSTM2A* (NM_001301009.1), VSTM2A IgV domain (Δ144-240), and VSTM2A Δ N (Δ1-25) were amplified and cloned into the pcDNA3.1 expression vector. Human LRP6 deletions (ΔE1-4, ΔE3-4, and ΔN) are kind gifts of Prof. Christof Niehrs. Human full length LRP6 and deletion (ΔE1-2 and ΔC) were generated by PCR and cloned into pcDNA3.1.

### Cell viability and colony formation assays

Cells were seeded for MTT and MTS assay to measure the cell viability. Cells were seeded on 6-well plates and stained with 0.5% crystal violet solution for 30 min. Colony with more than 50 cells per colony was counted. The experiment was conducted in three independent triplicates.

### Cell apoptosis by flow cytometry

Transfected cells were stained with Annexin V- Allophycocyanin (APC) (BD Biosciences) and 7-aminoactinomycin (7-AAD). The cells were sorted by FACS Calibur system.

### *In vivo* tumorigenicity

Stable VSTM2A expressing or control HCT116 cells (2×10^6^ cells in 0.1 ml phosphate-buffered saline) were injected subcutaneously into the right dorsal flank of four 4-week-old male Balb/c nude mice, separately (5/group). Tumor diameter was measured every 3 days. Tumor volume (mm^3^) was estimated by measuring the longest and shortest diameter of the tumor and calculating as follows: volume = (shortest diameter)^2^ × (longest diameter) × 0.5. After 15 days, the mice were sacrificed, and the tumors were weighed and fixed in formalin for histological analysis. All experimental procedures were approved by the Animal Ethics Committee of the Chinese University of Hong Kong.

### PCR analyses

Semi-quantitative PCR was performed by AmpliTaq Gold DNA polymerase (Thermo Fisher Scientific). Quantitative real-time PCR was performed by SYBR Green PCR Master Mix (Thermo Fisher Scientific) on 7500HT Fast Real-Time PCR System (Thermo Fisher Scientific). Primer sequences are listed in **Table S1**. Target gene expression was normalized to GAPDH expression and quantified using the delta-Ct method.

### Western blotting

Proteins were separated on 10-12% SDS-polyacrylamide gel electrophoresis and transferred onto Polyvinylidene difluoride membranes (GE Healthcare, Chicago, IL). Blots were immunostained with primary antibody and secondary antibody. Independent experiments were performed at least twice. The antibodies used and their dilutions are listed in **Table S2**.

### Immunohistochemistry (IHC) analysis and tissue microarray assay

Tissue microarrays of CRC tissues were stained with VSTM2A antibody. IHC was performed using the EnVision system (Agilent, Santa Clara, CA). The antibodies used and their dilutions are listed in **Table S2**. The results were scored independently by two pathologists.

### Co-immunoprecipitation (IP) and Mass Spectrometry Analysis

RKO cells were seeded on 10 X 100 mm Polyethylenimine (PEI, Sigma-aldrich) coated dishes. After 4 hours incubation with DMEM serum-free medium at 37^o^C, cells were chilled on ice followed by treatment of His-tag VSTM2A conditioned medium for 1 hour at 4^o^C. The cells were then incubated with 1.6 mM cell impermeable crosslinker 3,3'-dithiobis(sulfosuccinimidyl propionate (DTSSP, Sigma-Aldrich) for 2 h on ice. Whole-cell lysates were incubated with 400 μl Ni-NTA agarose for 12 h. Potential binding proteins were eluted with 1.5 ml RIPA buffer containing 250 mM imidazole and precipitated with 10% trichloroacetic acid. Sample was loaded to 10% SDS-PAGE for separation followed by Coomassie Blue staining. The gel was excised into 11 segments and submitted to Shotgun proteomics analyses using an EASY-nLCTM 1200 UHPLC system (Thermo Fisher) coupled with an Orbitrap Q Exactive HF-X mass spectrometer (Thermo Fisher) operating in the data-dependent acquisition (DDA) mode. The resulting spectra from each fraction were searched separately against Homo_sapiens_uniprot (169389 sequences) database by the search engines: Proteome Discoverer 2.2 (PD 2.2, Thermo).

### Titration ELISA

The Dissociation Constant between VSTM2A and LRP6 was determined by Titration ELISA 10. 5 ug/ml recombinant human LRP-6 Fc Chimera Protein (R&D, 1505-LR-025) was immobilized on ELISA plate (50 uL per well) at 4°C for 12 h. Coated plate was washed with PBS and blocked by 5% BSA solution in PBS for 12 h at 4°C. Recombinant Human VSTM2A His-tag Protein (R&D, 10037-VT) serial dilution (a start concentration of 100 ug/ml and dilution factor of 4) was incubated ELISA plate in duplicate for 2 h at room temperature. After remove non-bound VSTM2A, ELISA plate was washed twice with PBS. The protein interaction was fixed by DTSSP. LRP6-bound VSTM2A was determined by ELISA with Alkaline Phosphatase Yellow (pNPP) Liquid Substrate (Sigma) at OD405.

### Conditioned media and recombinant protein used in this study

Wnt3a-conditioned medium was harvested from Wnt3a expressing L-cells according to the manufacturer's instructions (ATCC). VSTM2A-conditioned medium and control medium were harvested from 293T cells transiently transfect with VSTM2A expression or empty control constructs. Briefly, 293T cells were seeded in 10 cm dishes and transfected with 16 ug of p3.1-VSTM2A-His/myc or p3.1 empty vector using Lipofectamine 2000 as directed by the manufacturer (Thermo Fisher Scientific). Conditioned medium was harvested 48 h after transfection. Recombinant human VSTM2A His-tag protein (10037-VT), LRP6 (1505-LR-025) and Wnt3a (5036-WN-010) were purchased from R&D.

### Internalization assay

Cells were labeled with EZ-Link Sulfo-NHS-SS-Biotin (company, city, country) prior to conditioned medium treatment to allow internalization of cell surface proteins performed as described previously 11. Biotinylated protein in the cell lysates was immunoprecipitated by Dynabeads® MyOne Streptavidin C1 (Thermo Fisher Scientific) and immunoblotted using anti-LRP6 antibody.

### Dual-luciferase reporter assay

Cells in a 24-well plate were co-transfected with luciferase reporter plasmid, and pRL-CMV control vector by lipofectamin 2000. Plasmids were co-transfected as indicated per well. After 24 h of transfection, luciferase activities were analyzed by Dual-Luciferase Reporter Assay System (Promega) and normalized to the control Renilla. The key cancer related signaling pathways including Wnt/β-catenin (TOPflash/FOPflash), nuclear factor-кB (NF-кB), MAPK (SRE), TGF-β (CAGA), JNK (AP-1), STAT3 (APRE), PI3K/AKT (FOXO), BMP (GCCG), and p53 (p53RE) were investigated.

### Treatment with 5-Aza and TSA

Cells were then treated with 2 μM demethylation reagent 5-aza-2'-deoxycytidine (5-Aza, Sigma-Aldrich) for 5 days. The 5-Aza was replenished every day. Some cell lines were further treated with 300 nM of histone deacetylase inhibitor trichostatin A (TSA, Sigma-Aldrich) for additional 24 h.

### Bisulfite genomic sequencing (BGS) analysis

Two μL bisulfite-treated DNA was amplified by primers of BGS and sequenced. Sequencing analysis was performed by SeqScape software (Thermo Fisher Scientific). Methylation percentage of each CpG site was calculated according this formula: Methylation% = H_C_/ (H_C_ + H_T_) × 100%, (H_C_ = Height of peak C, H_T_ = Height of peak T). Primer sequences for BGS are listed in Table S1.

### Statistical analyses

Values were expressed as mean ± standard deviation (SD). The Student's *t*-test was used to compare the difference between two groups. The χ2 test was used to compare the clinicopathologic characteristics of CRC patients and VSTM2A IHC scores. Univariate and multivariate Cox regression analysis were performed to assess the prognostic value of VSTM2A. Overall survival in relation to VSTM2A methylation status and IHC scores were evaluated by Kaplan-Meier survival curve and log-rank test. Differences with *P* value < 0.05 were considered to be statistically significant.

## RESULTS

### VSTM2A is commonly silenced in CRC patients

**Figure 1:**
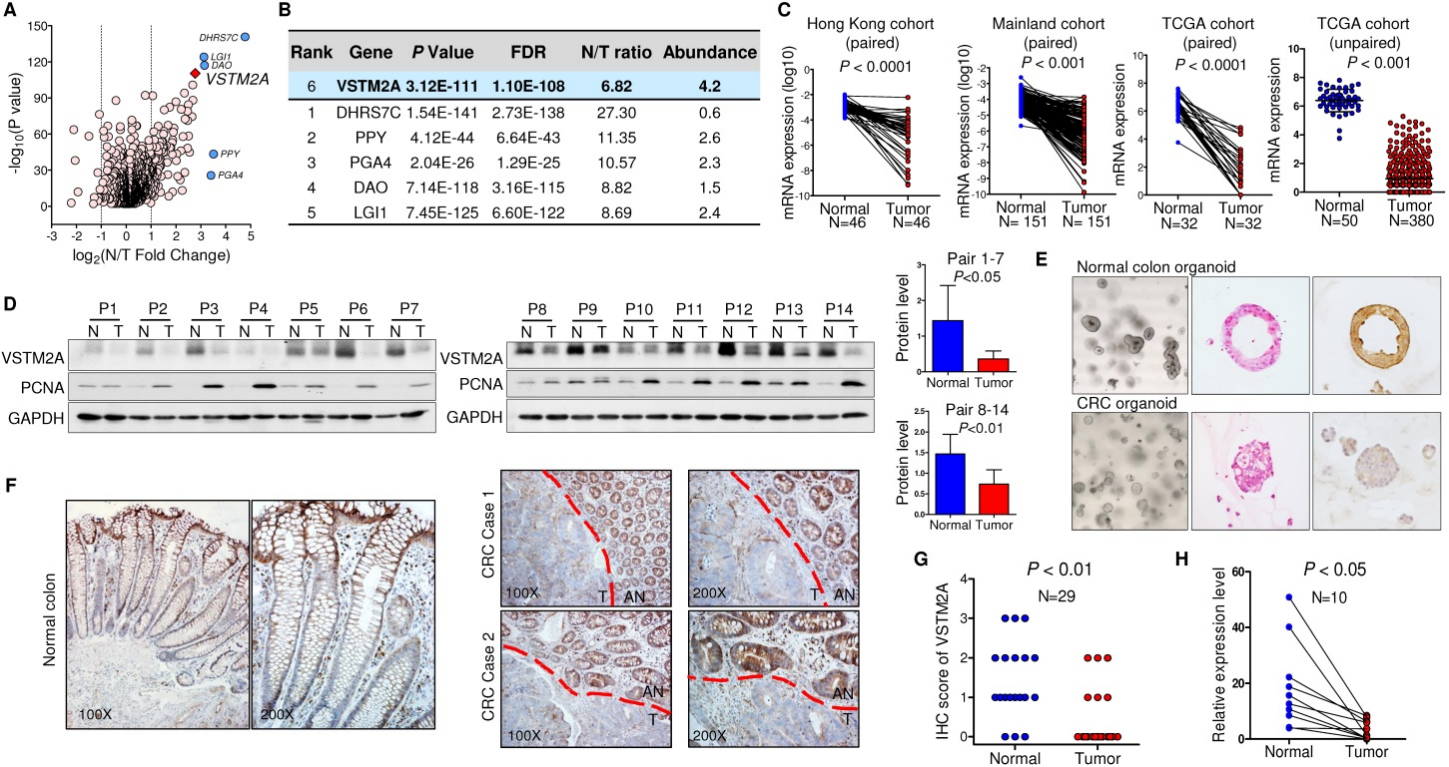
** VSTM2A is significantly down-regulated in human colorectal cancer. (A)** Volcano plot of dysregulated secreted protein and single-pass TM protein of TCGA CRC patients, with *P* values plotted versus the expression fold change. Both axis values are presented on a log scale with a vertical dotted lines indicate fold change = 2 and 1/2. VSTM2A is highlighted by red diamond. Genes discussed in (B) are highlighted by blue circles. **(B)** Top six downregulated secreted proteins in TCGA CRC patients. Candidate genes are ranked by normal vs. tumor fold change. **(C)** VSTM2A mRNA in primary tumor and matched adjacent nontumor tissue from CRC patients were determined by real-time PCR (Hong Kong cohort: N = 46, Mainland China cohort: N = 151). Expression data are shown following normalization for GAPDH. VSTM2A mRNA in TCGA CRC dataset was analyzed by paired and unpaired student T test. **(D)** VSTM2A protein in 14 pairs of randomly selected CRC cancer and adjacent normal tissues were determined by Western blot with GAPDH as internal control. Proliferating cell nuclear antigen (PCNA) serves as marker of cancerous counterpart in the Western blot. Quantitative analyses of VSTM2A protein by densitometry is shown in the right panel. **(E)** Representative photomicrographs showing H&E and IHC staining of VSTM2A in normal colon organoid and cancer organoid. **(F)** Representative photomicrographs showing IHC staining of VSTM2A in normal human colon tissue and primary CRC tissues under low power and high power magnification. Dashed red lines define zone of primary tumor tissue (T) and adjacent normal epithelia tissue (AN). **(G)** Statistical analysis of IHC staining of VSTM2A in 29 pairs of primary CRC tissues and adjacent normal tissues. **(H)** Real-time PCR analysis of VSTM2A expression in 10 randomly selected paired normal epithelia cell and primary tumor cells after elimination of stroma tissue using micro-dissection.

To identify the dysregulated secreted proteins, we adopted the list of candidate extracellular genes compiled by Gonzalez and colleagues, containing 4,642 unique human proteins encoded by 3,641 unique genes 9. A net of 1785 candidates were subject to TCGA gene expression analysis owing to their high expression in normal colorectal tissue (transcript per million (TPM) > 0.1) (Figure S1). 144 unique genes were significantly downregulated in colon cancer in comparison to the adjacent normal tissues (Figure 1A). We prioritized genes based on their fold change between adjacent normal/tumor tissue, and their abundance. Based on these two criteria, VSTM2A was identified as one of the top-downregulated genes with an average fold change of 6.82. It also shows a relatively high abundancy in normal colon tissue compared with the rest candidates, suggesting it may play an important role in maintenance of normal colon cell function (Figure 1B). We therefore selected it for further study.

We screened the VSTM2A expression status in normal human tissues and found that VSTM2A was predominantly expressed in gastrointestinal tract including colon (Figure S2). Compared to its readily expression in normal colon, VSTM2A mRNA expression was significantly reduced in CRC in two independent cohorts of 197 Chinese patients (Figure 1C). This was further confirmed in 398 CRC cases from TCGA cohort (Figure 1C). VSTM2A protein was also decreased in 14 randomly selected CRC tissues by Western blot (*P* < 0.05, Figure 1D). IHC staining showed that human-derived normal colon organoid showed a high expression of VSTM2A on the cell membrane compared with patient-derived colon cancer organoid (Figure 1E). Moreover, VSTM2A was prominently expressed in enterocytes and goblet cells, especially in the outermost layer of the crypt, whereas its expression was low or undetectable in Paneth-like cells localized at the bottom of the crypt and lamina muscularis mucosae by IHC (Figure 1F). Strong expression of VSTM2A was found in normal epithelium compared with paired tumor regions using IHC staining (Figure 1G). We eliminated the stromal tissue with micro-dissection in randomly selected 10 CRC cases. Consistently, VSTM2A mRNA was significantly decreased in tumor tissues compared with normal epithelial cells by real-time PCR (Figure 1H, *P* < 0.01).

### VSTM2A inhibits CRC cell growth, migration and invasion

**Figure 2:**
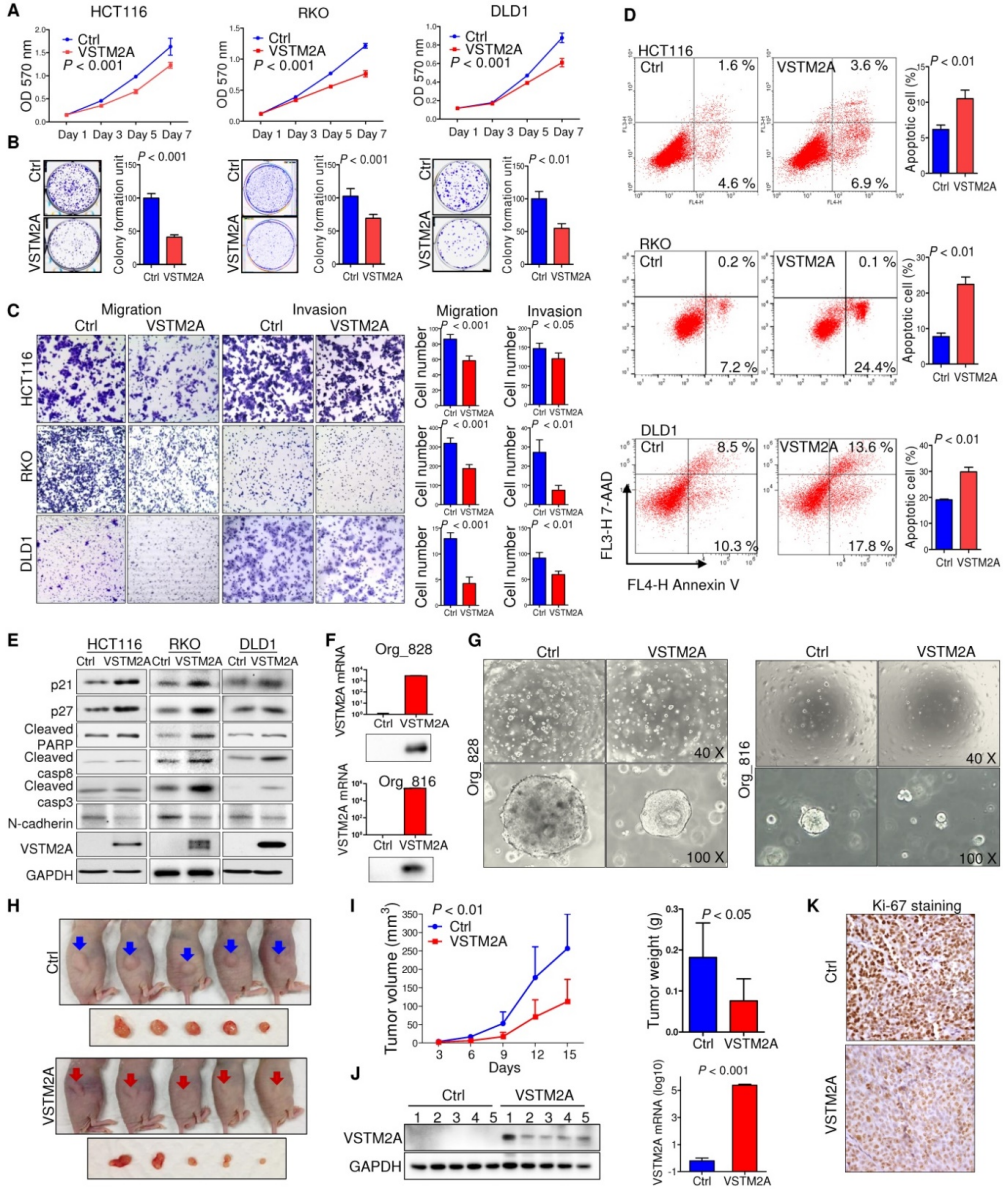
** VSTM2A inhibits CRC cell growth *in vitro* and *in vivo*. (A)** Effects of VSTM2A on CRC cell proliferation were evaluated by MTS assay. **(B)** Effect of VSTM2A on inhibition of colony formation of CRC cells. CRC cells transfected with VSTM2A or control plasmid were seeded into 6-well plates for a week. The amount of cell colonies was visualized by crystal violent staining. Data were normalized to control and expressed as percentage. **(C)** Effect of VSTM2A on metastatic ability of CRC cells using *in vitro* migration and invasion transwell assays. Statistical analysis was shown in the right panel. **(D)** Effects of VSTM2A overexpression on apoptosis were determined by flow cytometry analysis after the dual staining with Annexin V-APC and 7-AAD. Quantitative analysis of apoptotic cells (%) is shown in the right panel. **(E)** Protein levels of cell proliferation, apoptosis, and mesenchymal marker in CRC cells expressing VSTM2A were assessed by western blot analyses**. (F)** Overexpression of VSTM2A in two CRC organoid lines was determined by western-blot and real-time PCR. **(G)** Representative photomicrographs showed organoid number and size in control and VSTM2A overexpression groups. **(H)** Whole-body and gross tumor imaging of nude mice subcutaneously implanted HCT116 expressing control (ctrl) or VSTM2A plasmid. N = 5 mice per group. **(I)** Growth curves (left) and weight of xenograft tumours (right) derived from subcutaneously implanted HCT116 expressing control (ctrl) or VSTM2A plasmid. **(J)** VSTM2A protein and mRNA was determined when the tumors were harvested at day 15. **(K)** Representative IHC images of tissue sections from tumor xenografts stained for proliferation marker Ki-67, magnification x200. Data are plot by mean ± SD. Ctrl: empty vector without any gene expression.

Human *VSTM2A* has four transcripts that can generate protein products (http://www.ensembl.org). The four protein isoforms share an identical immunoglobulin V-set (IgV) domain but different protein sequences at the C terminal. Among these isoforms, *VSTM2A* isoform 4 (NM_001301009) showed the highest expression level in normal mucosal specimens, but reduced significantly in CRC (Figure S3A and S3B). We therefore selected *VSTM2A* isoform 4 for further functional studies. To characterize the biological role of VSTM2A, we ectopic expressed VSTM2A in three CRC cell lines (HCT116, RKO and DLD1). VSTM2A markedly reduced cell viability (Figure 2A) and colony formation ability (Figure 2B) in HCT116, RKO and DLD1 as compared with control cells. In addition, we observed that both overexpression of VSTM2A and recombinant VSTM2A protein significantly suppressed gastric cancer cell growth (Figure S4). We investigated the effect of VSTM2A on migration and invasion abilities of CRC cells using *in vitro* transwell with or without Matrigel matrix layer. Ectopic expression of VSTM2A in HCT116, RKO and DLD1 significantly suppressed cell migration and invasive capabilities (Figure 2C). In keeping with this, Western blot analyses showed that VSTM2A down-regulated mesenchymal marker (N-Cadherin) (Figure 2E).

We next investigated the effect of VSTM2A on cell apoptosis regulation by flow cytometry. ectopic expression of VSTM2A led to a significant increasing in apoptosis in HCT116 (*P* < 0.01), RKO (*P* < 0.01) and DLD1 (*P* < 0.01) by flow cytometry (Figure 2D), which was confirmed by increased protein expression of key cell apoptosis regulators cleaved caspase-3, cleaved caspase-8, and cleaved PARP in VSTM2A transfected cells (Figure 2E). These findings suggest that apoptosis induced by VSTM2A accounts for the CRC cell growth inhibition. We were unable to investigate the effects of VSTM2A knockdown since it was silenced in all the detected colon cell lines.

We validated the tumor suppressive role of VSTM2A in organoid models. Ectopic expression of VSTM2A by lentivirus transfection in two CRC patient-derived organoid models were confirmed by Western blot and real-time PCR (Figure 2F). Restoration of VSTM2A significantly reduced both organoid number and size (Figure 2G).

In light of our *in vitro* findings, we examined the *in vivo* tumor suppressive ability of VSTM2A. We observed a significant reduction in both xenograft size and mass in VSTM2A-expressing group compared with the control group (Figure 2H and 2I). VSTM2A expression in the xenografts was confirmed by western blot and real-time PCR (Figure 2J). VSTM2A expressing xenografts displayed significantly reduced cell proliferative activity compared to controls by Ki-67 immunostaining (Figure 2K). Collectively, our results suggest that VSTM2A plays a pivotal tumor suppressive role in CRC cells through inhibiting cell proliferation and promoting apoptosis.

### VSTM2A is a secreted protein antagonizing Wnt pathway

**Figure 3:**
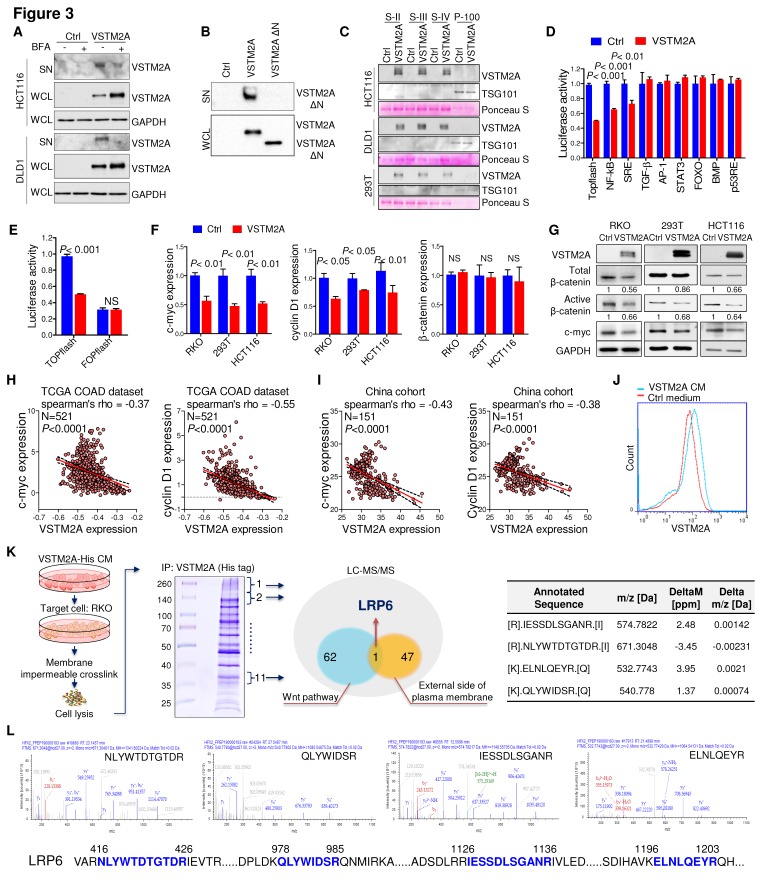
** Secreted protein VSTM2A binds to Wnt co-receptor LRP6 and inhibits Wnt signaling pathway. (A)** Flag-tagged VSTM2A was expressed in CRC cell lines. Cells were treated with either vehicle (DMSO) or Brefeldin A (10 ug/ml) for 1.5 h. Conditioned medium and cell pellets were blotted using indicated antibodies. **(B)** Immunoblot analysis of full length VSTM2A and N terminal deletion VSTM2A (Δ1-25) proteins in the culture supernatant (SN) and whole cell lysates (WCL). **(C)** Cell culture supernatants and exosomes were purified by ultracentrifugation. Supernatant fraction (S-II, S-III, S-IV) and 100,000 *g* pellet (P-100) were used for detecting of VSTM2A by Western blot analyses. TSG-101 serves as a marker of exosome. Ponceau S staining is used as a loading control. **(D)** Effects of VSTM2A on nine cancer related pathways were determined by luciferase reporter assay. 293T cell was co-transfected with either control plasmid or VSTM2A plasmid, in combination with each pathway luciferase reporter and pRL-CMV control reporter vectors for 24 h. The dual-luciferase assay was performed and results are expressed as fold change. **(E)** VSTM2A suppressed the Wnt signaling TOPflash reporter activity but not the FOPflash reporter. RKO cells were grown in 24-well plate and co-transfected with TOPflash or FOPflash plasmids. Normalized luciferase activity was measured 24 h after transfection.** (F)** Total RNA isolated from cell transfected with control and VSTM2A plasmid were subjected to Real-time PCR analysis. VSTM2A significantly suppressed c-myc and Cyclin D1 mRNA level in the indicated cell lines.** (G)** VSTM2A significant suppressed β-catenin activation analyzed in the indicated cell lines by Western blot analyses. Whole cell lysates were probed with the indicated antibodies. **(H)** Correlation between VSTM2A mRNA and Wnt target genes c-myc and cyclin D1 in the TCGA-COAD dataset. **(I)** Correlations between VSTM2A mRNA and Wnt target genes c-myc and cyclin D1 were validated in China CRC cohort using QPCR. **(J)** HCT116 cell was incubated with VSTM2A conditioned medium or control medium for 30 min at 37ºC and subjected to flow cytometry analysis of VSTM2A cell surface binding. **(K)** Left panel: experimental strategy for identification of the potential cell membrane receptor of VSTM2A by Ni-NTA purification and LC-MS/MS. Middle panel: coomassie blue-stained SDS-PAGE separation of VSTM2A-His tag binding proteins from RKO cells. Right panel: four peptide sequences are identified by LC-MS/MS analysis matched with human LRP6. **(L)** MS/MS spectra of the four peptides of LRP6. Ctrl: empty vector without any gene expression. CM: conditioned medium.

VSTM2A is predicted to have an N-terminal signal peptide (aa 1-25), suggestive of a secreted nature (http://www.uniprot.org/). Inhibition of the cell secretion by Brefeldin A (BFA), which prevents secreted protein transport from the endoplasmic reticulum (ER) to the Golgi apparatus, inhibited VSTM2A supernatant expression and induced cytosolic accumulation, suggesting a secreted property of VSTM2A (Figure 3A). Deletion the signal peptide of VSTM2A abolished its secretion into supernatant (Figure 3B), which is in consistent with previous study 6. VSTM2A was presented in the supernatant but not in the exosome fraction by sequential centrifuge steps and immunoblot analysis, suggesting that VSTM2A is released through a canonical secretion pathway (Figure 3C and Figure S5). To identify the molecular pathway mediated by VSTM2A, we screened the important cancer signaling pathways by luciferase reporter assays, including Wnt/β-catenin, NF-кB, MAPK, TGF-β, JNK, STAT3, PI3K/AKT, BMP, and p53. Among these pathways, Wnt/β-catenin signaling pathway was the top significantly suppressed pathway upon ectopic expression of VSTM2A (Figure 3D). VSTM2A suppressed the Wnt signaling TOPflash reporter activity down to 50% but not the FOPflash reporter in RKO cells (Figure 3E). Consistently, VSTM2A significantly inhibited the expression of Wnt target genes cyclin D1 and c-myc, and β-catenin activation, but did not change β-catenin mRNA expression in 293T and RKO cells, suggesting secreted protein VSTM2A negatively regulated Wnt signaling pathway in colon cancer (Figure 3F and 3G). It was reported that CRC cells carrying mutations of Wnt signaling (β-catenin and APC) can still response to the upstream ligands (Wnt3a and SFRP) 12, 13. We also observed that VSTM2A suppressed Wnt downstream targets in HCT116 (loss of β-catenin phosphorylation site S45) (Figure 3F). Moreover, VSTM2A mRNA level was negatively correlated with multiple Wnt target genes in the TCGA-COAD dataset, including c-myc, cyclin D1, AXIN2, BMP4, JUN, LGR5, MET and SOX9 (Figure 3H and Figure S6). The negative correlations between VSTM2A and c-myc /cyclin D1 were further validated in the China CRC cohort (Figure 3I).

### VSTM2A binds specifically to LRP6 on the cell plasma membrane

**Figure 4:**
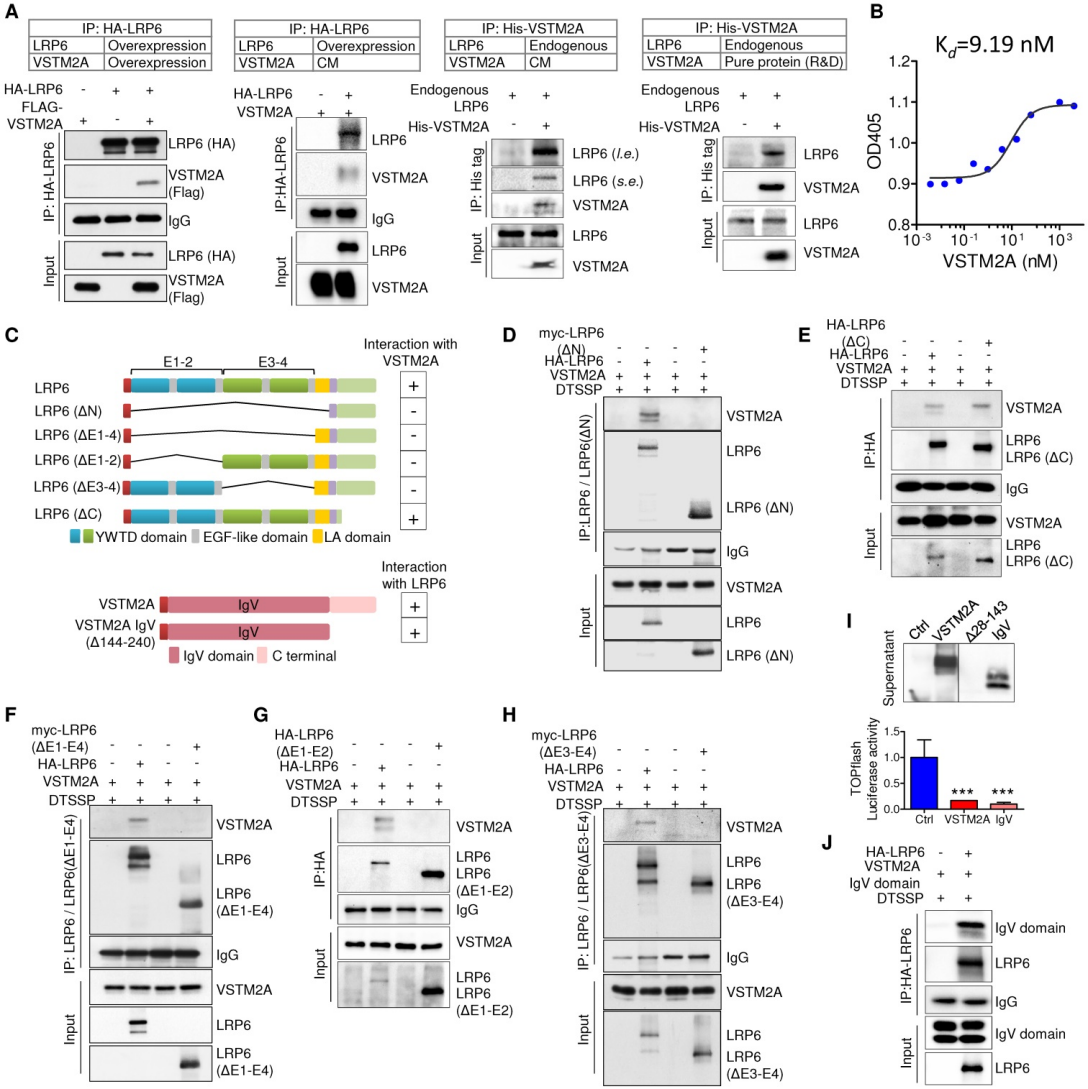
** VSTM2A binds to LRP6 E1-E4 domain with it IgV domain. (A)** Detection of the interaction between LRP6 and VSTM2A by co-IP.** (B)** Determine the dissociation constant of VSTM2A and LRP6 using tiltration ELISA. The ELISA was performed in duplicate. *K_d_* is 9.19 nM. **(C)** Schematic drawing of human full length LRP6 and deletions constructs employed, each with a HA or Myc tag (Upper panel). Schematic drawing of human VSTM2A and C terminal deletions employed, each with a signal peptide sequence on the N terminal and a Flag tag (Lower panel). Right panel, summary of the interaction properties of LRP6 and deletions with VSTM2A. **(D-H)** Domain mapping of LRP6 for VSTM2A binding. A series of LRP6 deletions were co-transfected with full length VSTM2A in 293T cells followed by treatment of 10 uM MG132 for 12 h. At 48 h after transfection, membrane protein was crosslinked, immunoprecipitated and analyzed by Western blot with the indicated antibodies. **(I)** VSTM2A full length, IgV set domain deletion (Δ28-143) and IgV domain expression in the supernatant was determined by western-blot. Effects of VSTM2A full length and IgV domain on Wnt signaling pathways were determined by TOPflash luciferase reporter assay. **(J)** Immunoprecipitation and immunoblot analysis of the interaction of full length LRP6 and VSTM2A C terminal deletion. CM, conditioned medium; *s.e.*, short-time exposure; *l. e.*, long-time exposure. Each Western blot experiment was repeated at least twice.

In an effort to characterize its cell surface receptor, HCT116 cells were exposed to VSTM2A conditioned medium generated from VSTM2A-stable expressing 293T cells. As shown in Figure 3J, VSTM2A attached to cell plasma membrane by immunofluorescence signals from Flow Cytometry analysis. To identify the potential receptor of VSTM2A on the cell plasma membrane, His-tagged VSTM2A conditioned medium were incubated with RKO cell. Whole cell lysis was immunoprecipitated with Ni-NTA beads followed by LC-MS/MS Analysis analysis (Figure 3K). 47 proteins located external side of plasma membrane identified by LC-MS/MS. We screened the potential candidate receptor that regulate Wnt pathway. LDL receptor related protein 6 (LRP6) was identified as a potential interacting protein with VSTM2A (Figure 3K and 3L, Table S3). LRP6 is a transmembrane Wnt co-receptor essential for Wnt/β-catenin signaling 14. To confirm the LC-MS/MS result, we performed co-immunoprecipitation (Co-IP) on 293T cells overexpressing of both Flag-tagged VSTM2A and HA-tagged LRP6. As shown in Figure 4A (the first panel), Co-IP using the enriched membrane-related protein fraction with an anti-HA antibody showed the interaction of VSTM2A and LRP6. We further purified VSTM2A protein from 293T cell culture supernatant and incubated with cell plasma membrane fraction containing HA-LRP6 protein. The interaction of LRP6 and VSTM2A was confirmed using anti-HA tag antibody (Figure 4A, the second panel). The interaction between LRP6 and VSTM2A was also validated by reciprocal IP using VSTM2A conditioned medium and endogenous LRP6 (Figure 4A, the third panel), and by reciprocal IP using recombinant human VSTM2A His-tag and endogenous LRP6 (Figure 4A, the fourth panel). Furthermore, we determined the VSTM2A and LRP6 protein dissociation constant (K*d*) using titration ELISA. The K*d* of recombinant human LRP-6 Fc chimera protein and recombinant human VSTM2A His-tag protein is 9.19 nM (Figure 4B). These results suggested that VSTM2A interacts with the cell surface receptor LRP6 in CRC cells.

### LRP6 E1-E4 domain is critical for its binding to VSTM2A IgV domain

The LRP6 extracellular domains consist of four YWTD-type β-propeller domain and four EGF-like domain pairs and one LDLR type A (LA) domain 15. For simplicity, the four “YWTD-type β-propeller and EGF pair” were referred as “E1”, “E2”, “E3” and “E4” (Figure 4C). To determine VSTM2A binding to which extracellular domain of LRP6, we analyzed the binding abilities of VSTM2A with various LRP6 deletions: LRP6ΔC (lacking the carboxyl cytoplasmic tail), LRP6ΔN (lacking all essential extracellular domain but anchored on the membrane), LRP6ΔE1-E4, LRP6ΔE1-E2 and LRP6ΔE3-E4 (Figure 4C). We found that the longest deletion LRP6ΔN lost binding ability with VSTM2A (Figure 4D), whereas LRP6ΔC which lacking the carboxyl cytoplasmic tail showed a strong binding (Figure 4E). Interestingly, LRP6 ΔE1-4, lacking all the four YWTD-type β-propeller domain and EGF-like domain but preserved the LA domain, was unable to recognize VSTM2A, indicating that the LA domain is dispensable for interacting with VSTM2A (Figure 4F). Deletion LRP6ΔE1-2 abolished the binding ability (Figure 4G). A similar result was observed using the deletion LRP6ΔE3-4, indicating that E1-4 domains are necessary for interacting with VSTM2A (Figure 4H).

VSTM2A protein contains a potential 25-aa N-terminal signal peptide and a single IgV domain spanning from aa 33-143 (Figure 4C). Despite the RNA level was well expressed, the VSTM2A IgV set domain deletion (Δ28-143) cannot be detected by western blot and therefore cannot be used for detecting the interaction with LRP6 (Figure 4I). Interestingly, VSTM2A Δ144-240, lacking the C terminal but well preserving the IgV domain, showed a strong binding with full length LRP6 and significantly suppressed TOPflash activity, indicating that the IgV domain is critical for the interaction with LRP6 (Figure 4I and 4J). Collectively, our results indicate that secreted protein VSTM2A bind to LRP6 E1-4 domain through its IgV domain.

### VSTM2A inhibits Wnt signaling through reducing LRP6 activity

**Figure 5:**
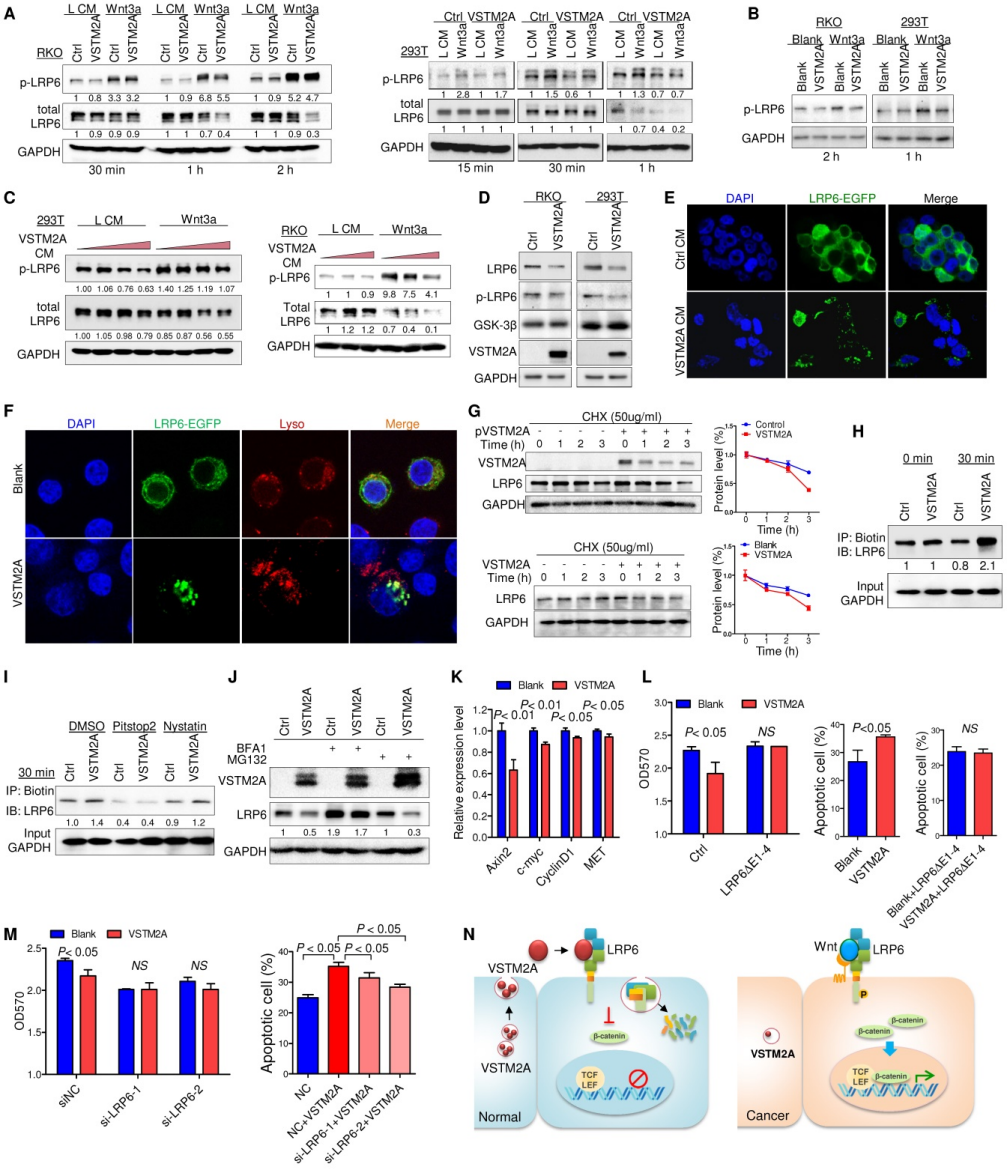
** VSTM2A suppresses LRP6 phosphorylation and induces LRP6 degradation. (A)** After RKO and 293T cells had been starved overnight, the cells were stimulated with the combination of Wnt3a and VSTM2A conditioned mediums (CM) for the indicated period of time. Whole cell lysates were probed with the indicated antibodies. **(B)** RKO and 293T cells had been starved overnight. Cells were stimulated with the combination of recombinant Wnt3a protein and recombinant VSTM2A protein for the indicated period of time. Whole cell lysates were probed with the indicated antibodies. **(C)** RKO and 293T cells were starved overnight and stimulated with L or Wnt3a CM in the presence of increased dose of VSTM2A CM for 1 h. Whole cell lysates were probed with the indicated antibodies.** (D)** Ectopic expression of VSTM2A reduced LRP6 protein level in RKO and 293T cells. Whole cell lysates were probed with the indicated antibodies. **(E)** Representative images of LRP6-EGFP localization. 293T cells expressing LRP6-EGFP were stimulated with VSTM2A conditioned medium or control medium for 1 h at 37°C, and the fixed cells was viewed directly. **(F)** Representative images of LRP6-EGFP localization. RKO cells expressing LRP6-EGFP were stimulated with VSTM2A pure protein or PBS for 1 h at 37°C, and the fixed cells was viewed directly. LysoTracker™ was used for staining of lysosome. **(G)** Upper panel: 293T cells were transfected with control or VSTM2A plasmid for 48 h. Lower panel: RKO cells were treated with recombinant human VSTM2A for 1h, 2h, 3h in serum free medium. Protein synthesis was inhibited by 50 ug/ml CHX for the indicated times. Half-life of endogenous LRP6 protein was determined by Western blot analysis. **(H)** 293T cell surface proteins were biotinylated using a reversible biotinylation agent Sulfo-NHS-SS-Biotin. Following labelling, cells were stimulated with VSTM2A conditioned medium or control medium for the indicated times at 37°C. Cell was treated with sodium 2-mercaptoethanesulfonate to strip away any biotinylated proteins remaining on the cell surface. Whole cell lysis was precipitated with Dynabeads® MyOne™ Streptavidin C1 followed by immunoblot analysis with anti LRP6 antibody. VSTM2A conditioned medium induced cell surface LRP6 biotinylation (internalization) at 30 min. **(I)** VSTM2A conditioned medium induced cell surface LRP6 internalization was suppressed in the absence of clathrin-mediated endocytosis inhibitor Pitstop® 2 (30 μm) but not caveolin-mediated endocytosis inhibitor Nystatin (40 μm). **(J)** 293T cells expressing VSTM2A or empty vector was treated with Bafilomycin A1 (100 nM) and MG132 (10 μM) for 8 h. Whole cell lysates were probed with the indicated antibodies.** (K)** RKO was treated with VSTM2A recombinant protein or PBS for 24 hous. Wnt target genes were quantified by QPCR.** (L)** LRP6(ΔE1-4) was overexpressed in RKO followed by treatment with recombinant human VSTM2A His-tag protein (10 ug/ml). Cell growth was measured by MTT assay. Cell apoptosis was measure by flow cytometry. **(M)** RKO endogenous LRP6 was silenced by siRNA in the presence or absence of VSTM2A protein. Cell growth was measured by MTT assay. Cell apoptosis was measure by flow cytometry. **(N)** Proposed mechanistic scheme of secreted protein VSTM2A inducing LRP6 degradation and suppressing the Wnt signaling pathway in colorectal cancer. Each Western blot experiment was repeated at least twice.

VSTM2A interaction with LRP6 might initiate an intracellular signal responsible for Wnt inhibition at a level upstream of the β-catenin degradation complex, as Wnt signaling is initiated when Wnt proteins interacted with the co-receptor LRP6 and then transduction of the Wnt signaling inside the cell through phosphorylation of the intercellular domain of LRP6 16. To gain insights into the molecular mechanisms underlying Wnt/β-catenin signaling regulatory action of VSTM2A, we investigated whether LRP6 phosphorylation was inhibited by VSTM2A. RKO and 293T cells were used in the following assays due to their high expression level of endogenous LRP6 and low basal level of activated Wnt signaling. After starvation, cells were treated with VSTM2A in a combination with L medium or Wnt3a conditioned medium for different lengths of time, and cell extracts were analyzed by Western blot. Wnt3a stimulation robustly catalyzed LRP6 (Ser1490) phosphorylation, while VSTM2A conditioned medium suppressed the ability of Wnt3a to induce LRP6 phosphorylation (Figure 5A). We treated 293T and RKO with recombinant VSTM2A and recombinant Wnt3a. Consistently, LRP6 phosphorylation induced by Wnt3a was inhibited by VSTM2A protein in both cell lines (Figure 5B). Moreover, VSTM2A conditioned medium inhibited Wnt3a mediated transactivation of LRP6 and suppressed LRP6 protein expression in a dose-dependent fashion (Figure 5C). We also observed that VSTM2A conditioned medium reduced the LRP6 total protein level in both cell lines (Figure 5A and 5C). In keeping with the effect of VSTM2A conditioned medium, ectopic expression of VSTM2A in RKO and 293T significant reduced LRP6 protein level without affect its mRNA (Figure 5D and Figure S7).

### VSTM2A induces LRP6 endocytosis and concomitantly degradation

In particular, VSTM2A conditioned medium caused the internalization of LRP6-EGFP in 293T cells, whereas majority of LRP6-EGFP in 293T cells was retained on the cell surface membrane when treated with control medium (Figure 5E). Furthermore, recombinant VSTM2A protein significantly induced LRP6 endocytosis and co-localization with acidic organelles in RKO cells (Figure 5F). We measured the half-life of endogenous LRP6 following inhibition of new protein synthesis by cyclohexamide (CHX). Indeed, the half-life of LRP6 was significantly decreased in VSTM2A-transfected compared with the control group within 3 hours (Figure 5G, upper panel). A similar trend was observed by using VSTM2A recombinant protein (Figure 5G, lower panel). To confirm the effects of VSTM2A on LRP6 endocytosis and degradation, we labelled cell surface proteins with a reversible biotinylation agent (Sulfo-NHS-SS-Biotin) and treated with VSTM2A conditioned medium for 30 min (Figure S8). VSTM2A significantly increased the biotin labelled LRP6 at 30 min, confirming VSTM2A induced LRP6 internalization (Figure 5H). We next treated 293T cell with a selective inhibitor for clathrin-mediated endocytosis (Pitstop®2), or a selective inhibitor of caveolae-mediated endocytosis (Nystatin), prior exposure to VSTM2A conditioned medium. As shown in Figure 5I, VSTM2A induced a clathrin-mediated endocytosis of LRP6 rather than caveolae-mediated endocytosis by internalization assay. Given that VSTM2A conditioned medium induced LRP6 internalization and degradation, we examined if LRP6 degraded by lysosome or proteasome. 293T cell were treated with lysosome inhibitor Bafilomycin A1 and proteasome inhibitor MG132. Bafilomycin A1, but not MG132, blocked VSTM2A-induced degradation of LRP6 (Figure 5J).

VSTM2A protein significantly suppressed Wnt downstream targets (Axin2, c-myc, cyclin D1 and MET) mRNA expression (Figure 5K). Overexpression of LRP6(ΔE1-4) abolished VSTM2A protein effects on cell proliferation inhibition and pro-apoptosis (Figure 5L). Consistently, silencing of LRP6 significantly abolished the proliferation inhibition effect and undermined the pro-apoptotic effect of VSTM2A (Figure 5M and Figure S9). Collectively, our study suggested that VSTM2A caused LRP6 protein endocytosis and consequent lysosome mediated degradation (Figure 5N).

### VSTM2A is associated with the survival of CRC patients

**Figure 6:**
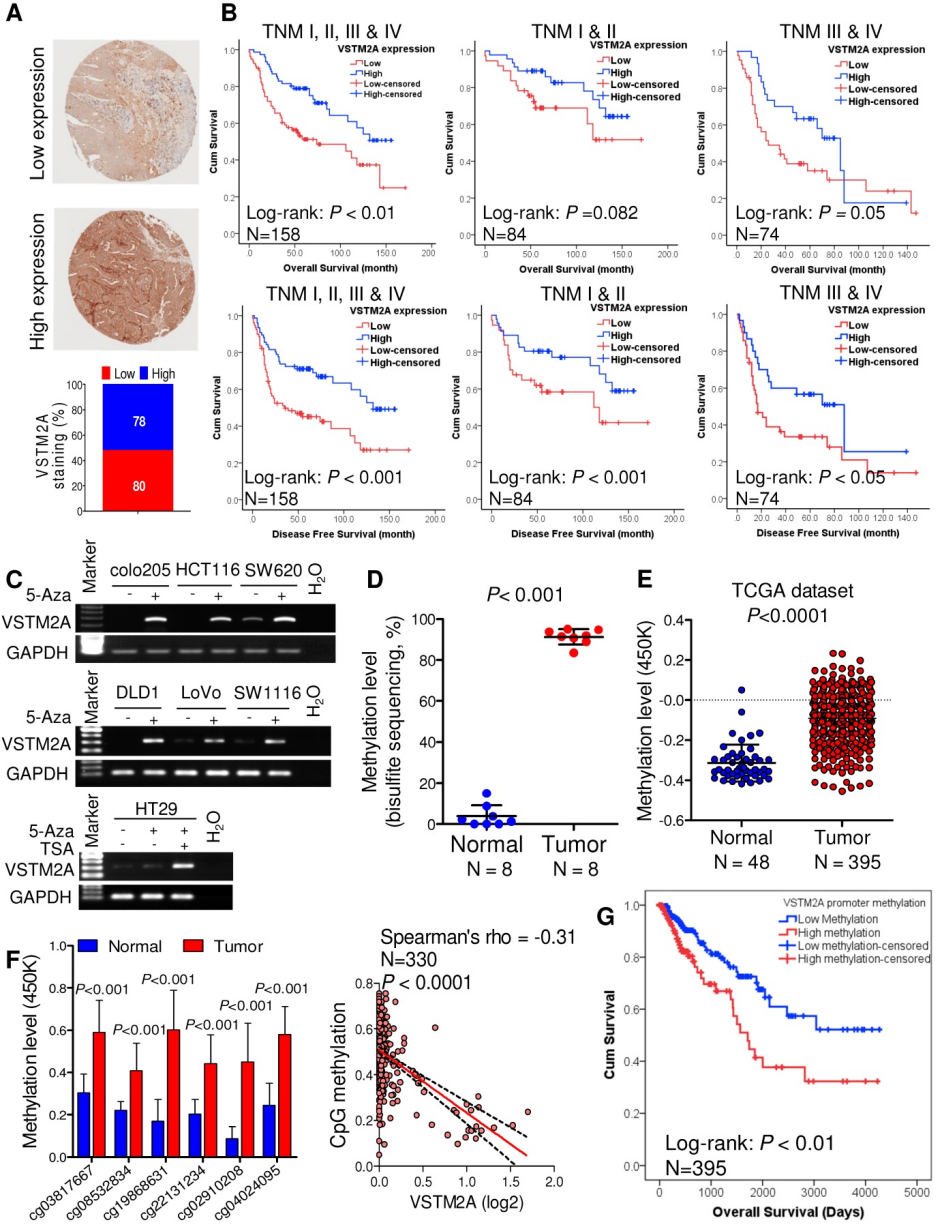
** VSTM2A is associated with the survival of CRC patients. (A)** IHC staining of VSTM2A in colorectal tissue microarray (TMA) (N = 158). Samples are graded as low and high expression, with representative histology shown. Magnification: x100. **(B)** Kaplan-Meier plot of overall survival and disease-free survival for 158 CRC patients stratified based on either low (N = 80) or high (N = 78) VSTM2A IHC staining scores. Patients with high expression of VSTM2A tumors showed a statistically significant higher overall survival and disease-free survival probability (log-rank test, *P* < 0.01 and *P* < 0.001). **(C)** Re-expression of VSTM2A in seven CRC cell lines following 2 μM 5-Aza or 300 nM TSA treatment. Expression levels of VSTM2A were determined by semi-quantitative PCR. Treatment of demethylating agent 5-Aza and histone deacetylase inhibitor TSA restored VSTM2A expression in CRC cell lines. **(D)** Quantitative analyses of VSTM2A CpG islands 15-40 in 10 primary tumors and matched adjacent nontumor tissues from CRC patients by BGS assay. **(E)** The average value of CpG probes located at VSTM2A promoter region (cg03817667, cg08532834, cg22131234, cg15164958, cg19868631, cg21629006, cg25528143, cg04024095 and cg02910208) in tumor and normal tissues from TCGA dataset was analyzed by unpaired student T test. **(F)** Six CpG probes located at VSTM2A promoter region was increase in tumor tissue and negatively associate with VSTM2A mRNA. **(G)** Kaplan-Meier survival plot of TCGA CRC patients stratified by VSTM2A promoter methylation status was used for the log-rank test.

To explore the clinical relevance of VSTM2A, we evaluated the VSTM2A protein expression in tissue microarray including 158 CRC patients and correlated with their clinical features. Tumor samples were stratified into high and low expression groups based on VSTM2A staining assessed by two independent pathologists (Figure 6A). No correlations were found between VSTM2A expression and clinicopathological features such as age, gender, differentiation, primary cancer location and Tumor-Nodes-Metastasis (TNM) stage (Table S4). Kaplan-Meier survival curves indicated that low VSTM2A staining was significantly correlated with shorter overall survival and disease free survival (Figure 6B). After adjustment for potential confounding factors, VSTM2A low expression was found to be an independent risk factor for shortened overall survival (RR 1.75, 95% CI 1.03 - 2.95, *P* = 0.04) and disease free survival (RR 1.86, 95% CI 1.14 - 3.05, *P* = 0.014) by multivariate Cox regression analysis (Table 1).

We investigated the mechanism of VSTM2A downregulation in CRC. We found full or partial methylation in all seven CRC cell lines (colo205, DLD1, HCT116, HT29, LoVo, SW620, and SW1116); whilst no or low methylation was detected in the four normal colon tissues (Figure S10). Demethylation by 5-Aza and TSA restored VSTM2A expression in all silenced cell lines (Figure 6C). We also observed that 5-Aza/TSA treatment upregulated LRP6 mRNA and Wnt downstream targets expression (Figure S11). VSTM2A promoter methylation was significantly higher in tumor tissues compared with the normal colon tissues in our cohort (N = 8, *P* < 0.001, Figure 6D and Figure S12) and in TCGA-COAD DNA Methylation Array Methylation450k cohort (N = 395, *P* < 0.0001, Figure 6E). We further identified six CpG probes located in promoter and 5'UTR region were negatively correlated with VSTM2A mRNA expression, suggesting VSTM2A silencing in CRC is mainly mediated by promoter hypermethylation (Figure 6F and Figure S13). Moreover, CRC patients with high VSTM2A promoter methylation had significantly worse overall survival than those with low or no methylation based on the log-rank test (Figure 6G, *P* < 0.01). After adjustment for potential confounding factors, lower VSTM2A methylation was found to be an independent risk factor for overall survival in CRC patients by multivariate Cox regression analysis (RR 1.67 (95% CI 1.01 to 2.73), *P* = 0.044; Table S5).

## DISCUSSION

Secreted proteins not only participate in various physiological processes but also play crucial roles in carcinogenesis 17. Approximately 30% of the human genome are predicted to be secreted proteins, making it a rich source for identification of crucial players of cancer development. However, the function of the majority of these proteins is still largely unknown. Therefore, understanding the molecular signaling pathways of secreted protein involved in carcinogenesis will provide basis for potential treatment regimens and new candidate cancer biomarkers identification. In this study, we first identified that VSTM2A was one of the top downregulated secreted protein in CRC. Using a series of *in vitro* and *in vivo* functional experiments, VSTM2A possesses a tumor-suppressive function through inhibiting CRC cell proliferation, migration/invasion, and organoid growth, induced apoptosis *in vitro* and suppressed xenograft tumor growth in nude mice.

We revealed that VSTM2A attached to plasma membrane and interacted with plasma membrane protein LRP6 extracellular domain E1-E4 by LC MS/MS analysis and immunoprecipitation. Unlike the groups of Wnt antagonist of the SFRPs which suppressed the Wnt signaling through indirectly sequestering Wnt proteins, VSTM2A directly binds to the Wnt receptor LRP6, which is functionally more similar to the Wnt inhibitor DKKs family 18. Same as DKKs, orthologs of VSTM2A was found in vertebrate such as Zebrafish and Xenopus but appear to be absent in *Drosophila* or *C. elegans*. However, no sequence similarity was found between DKKs and VSTM2A, suggesting a novel suppressor VSTM2A for LRP6. We measured that K*d* of human LRP-6 Fc and VSTM2A His-tag protein is 9.19 nM by titration ELISA, which is lower than that of DKK1 (0.35-0.84 nM measured by surface plasmon resonance technology, SPR) 19. Accurate analysis of *Kd* by titration ELISA requires pure preparations of both receptor and ligand. Considering the 80% purity of commercial available LRP-6 Fc, the K*d* of LRP-6 Fc and VSTM2A should be further investigated by a cost-intensive SPR machine. Moreover, we observed that overexpression of VSTM2A showed double bands in western blot analysis as showed in Figure 3G and Figure S14. The double bands may be caused by post modification of the VSTM2A. Whether the post-modification affects the interaction between VSTM2A and LRP6 needs further investigation. Taken together, our results reveal that Wnt inhibitor VSTM2A binds with LRP6 extracellular E1-E4 domain via its IgV domain which is functionally important for inhibiting LRP6 protein level. Loss of VSTM2A may cause Wnt signaling hyper-activation in colon and finally lead to cancer development.

LRP6 is a critical co-receptor of Wnt ligands.^3^, ^14^ In the presence of canonical Wnt ligands, LRP6 is phosphorylated by CK1γ and GSK3β serves as a platform for translocation of Axin, a member of the destruction complex, to the plasma membrane. Subsequently, cytoplasmic β-catenin is stabilized and forms a transcriptionally active complex with LEF/TCF in the nucleus, thereby activating expression of various genes 3, 14. LRP6 phosphorylation is an indispensable step for routing the canonical Wnt signal pathway. Following binding with LRP6, VSTM2A significantly inhibited LRP6 phosphorylation in a dose and time dependent manner. The effect of VSTM2A inhibiting LRP6 phosphorylation was more pronounced in the presence of Wnt agonist Wnt3a. Most proteins that antagonize ligand-receptor interactions act through molecular mimicry to inhibit ligand binding to the receptor competitively. These findings suggested that VSTM2A may compete with Wnt3a on binding with LRP6 and suppressing it activation.

Ligand binding with receptor often causes the receptor endocytosis 14. We observed that VSTM2A induced faster and stronger LRP6 reduction in 293T compared with in RKO cells. This may cause by the different endocytosis rate/speed of different cell lines. Therefore we used different time points setting for the two cell lines in the time course experiment. Immunofluorescence assay further indicated that VSTM2A reduced cell surface level of LRP6 and promoted its endocytosis with 1 h of exposure. Clathrin- and caveolae-mediated endocytosis are two major endocytosis pathways that have been implicated in LRP6 signaling. Previous studies indicated that Wnt ligands induce caveolin-dependent endocytosis of LRP6 to relay signal downstream, whereas antagonists such as DKKs promote clathrin-dependent endocytosis, leading to inhibition 18. Pitstop® 2 and Nystatin are small molecule inhibitors selectively inhibit clathrin- and caveolae-mediated endocytosis respectively. Pitstop® 2 significantly diminished VSTM2A induced LRP6 endocytosis, whereas Nystatin had no such effect. These results provide further evidence that VSTM2A induced LRP6 endocytosis via a clathrin-mediated mechanism. These observations point toward a function for VSTM2A in regulating of LRP6 degradation. Indeed, LRP6 levels was significantly restored by suppression of lysosome-mediated degradation, but were unaffected by inhibition of the proteasomal degradation pathway. Collectively, our results demonstrate that VSTM2A is a novel negative regulator of Wnt pathway by direct binding with LRP6, inhibiting its phosphorylation and in particular, causing LRP6 endocytosis and lysosome mediated degradation (Figure 5N).

In summary, we revealed that secreted protein VSTM2A is a critical tumor suppressor in colorectal carcinogenesis and a novel antagonist of Wnt signaling receptor LRP6. VSTM2A suppresses Wnt/β-catenin signaling by directly inhibiting LRP6 activity and inducing LRP6 endocytosis and lysosome mediated degradation. VSTM2A may serve as a new prognostic marker for CRC patients.

## Figures and Tables

**Table 1:**
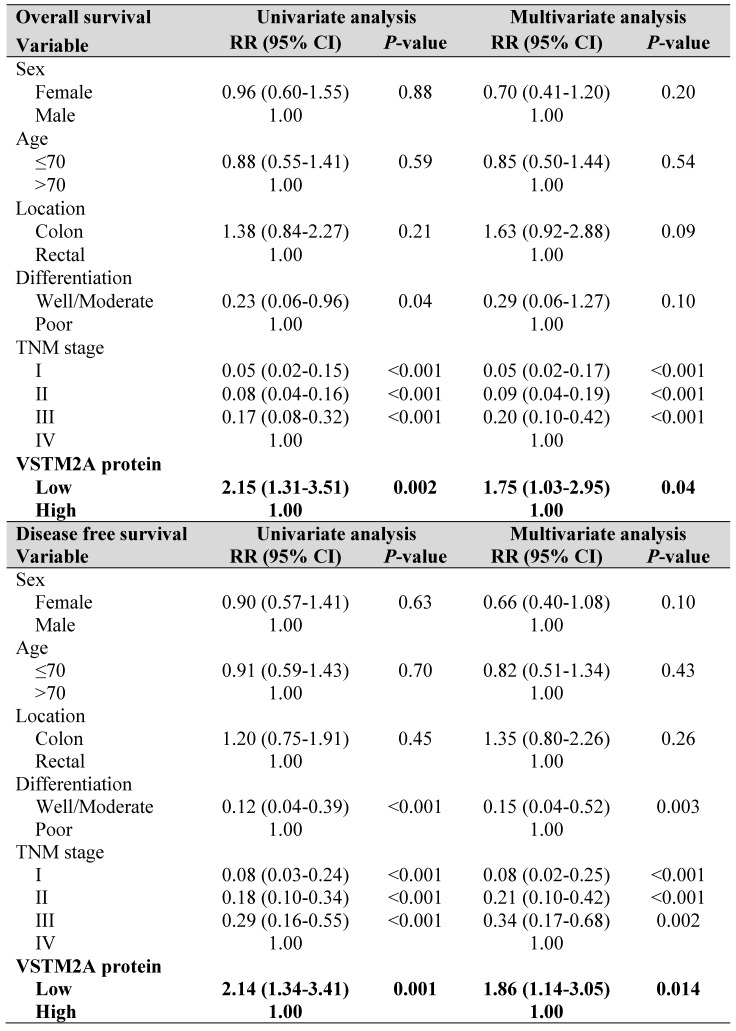
Univariate and multivariate Cox regression proportional hazards analysis for overall survival and disease free survival in Hong Kong CRC patients (N = 158).

## Abbreviations

5-Aza: 5-aza-2'-deoxycytidine
7-AAD: 7-aminoactinomycin
aa: amino acid
APC: V- Allophycocyanin
BFA: Brefeldin A
BGS: Bisulfite genomic sequencing
CI: confidence interval
COADREAD: colon & rectum adenocarcinoma
CRC: colorectal cancer
DKK: Dickkopf
DTSSP: 3,3'-dithiobis(sulfosuccinimidyl propionate
ER: endoplasmic reticulum
FACS: fluorescence activated cell sorting
IHC: Immunohistochemistry
LRP6: LDL receptor related protein 6
MTS: 3-(4,5-dimethylthiazol-2-yl)-5-(3-carboxymethoxyphenyl)-2-(4-sulfophenyl)-2H-tetrazolium
RR: Relative Risk
SFRP: secreted frizzled-related protein
TCGA: The Cancer Genome Atlas
TNM: Tumor-Nodes-Metastasis
TPM: transcript per million
TSA: trichostatin A
VSTM2A: V-set and transmembrane domain containing 2A
