# Supplementary material for: VSTM2A suppresses colorectal cancer and antagonizes Wnt signaling receptor LRP6
Source: Theranostics. 2019 Aug 21;9(22):6517–31. doi: 10.7150/thno.34989 (PMC6771244; doi:10.7150/thno.34989)
Supplement: Supplementary file 1 — Supplementary figures and tables. [file thnov09p6517s1.pdf]

Supplementary Figure

Figure S1. Schematic illustration of the workflow for identification of VSTM2A as one of the most significant downregulated secretory protein.

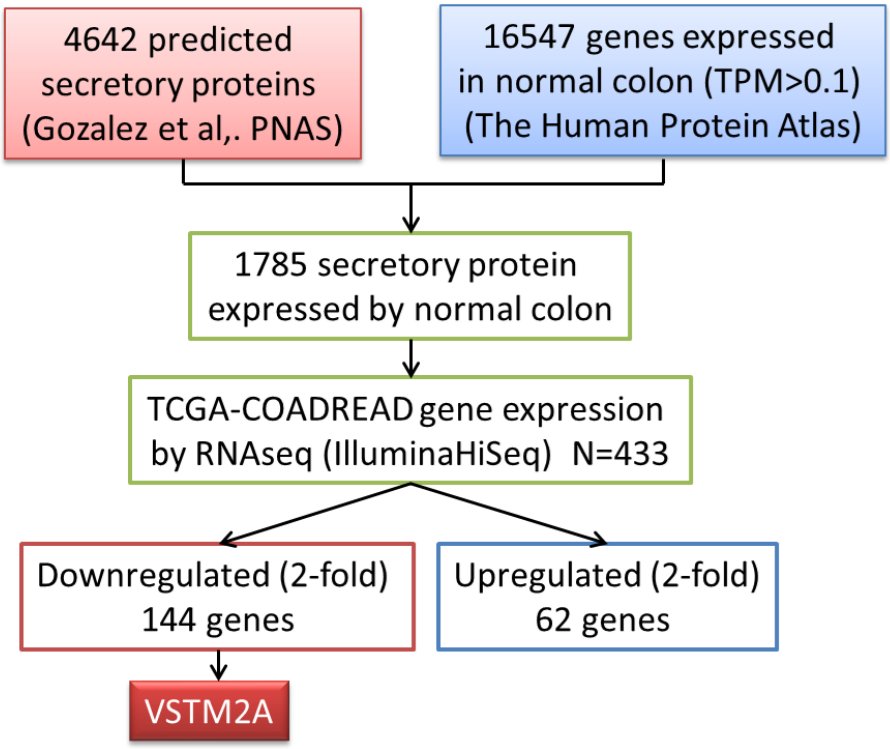

Figure S2. Semi-PCR analysis of VSTM2A expression in 21 human organs.

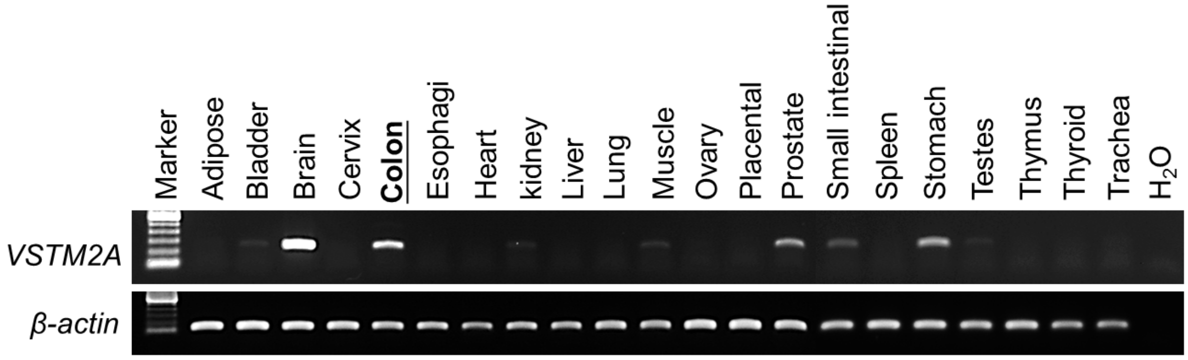

Figure S3. *VSTM2A* isoforms expression in normal colon and CRC. (**A**) 4 *VSTM2A* isoforms expression in normal colon tissues. (**B**) 4 *VSTM2A* isoforms expression in paired tumor and adjacent normal tissues.

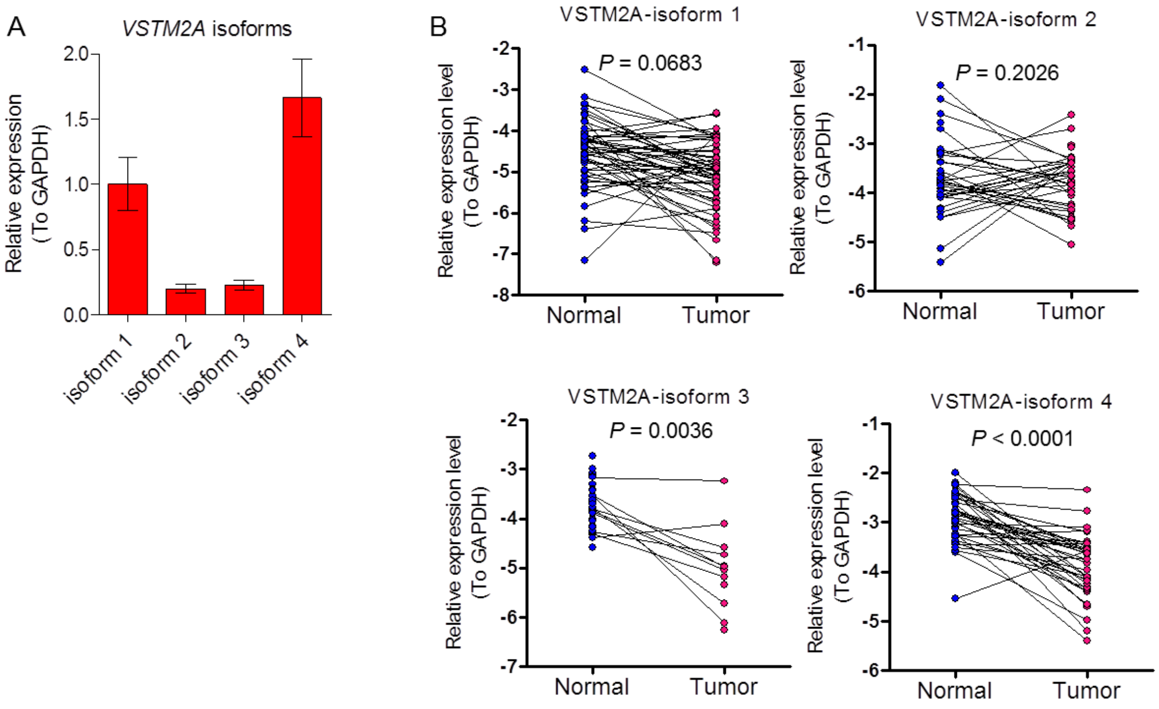

Figure S4. Overexpression of VSTM2A and treatment with recombinant VSTM2A protein significantly suppressed gastric cancer AGS and MGC803 cell growth by MTT assay.

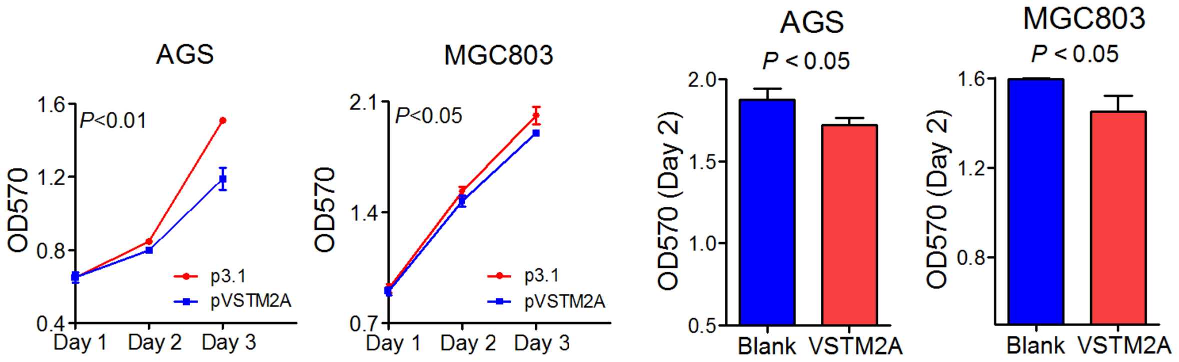

Figure S5. Differential centrifugation protocol for the isolation of exosomes from cell culture supernatants.

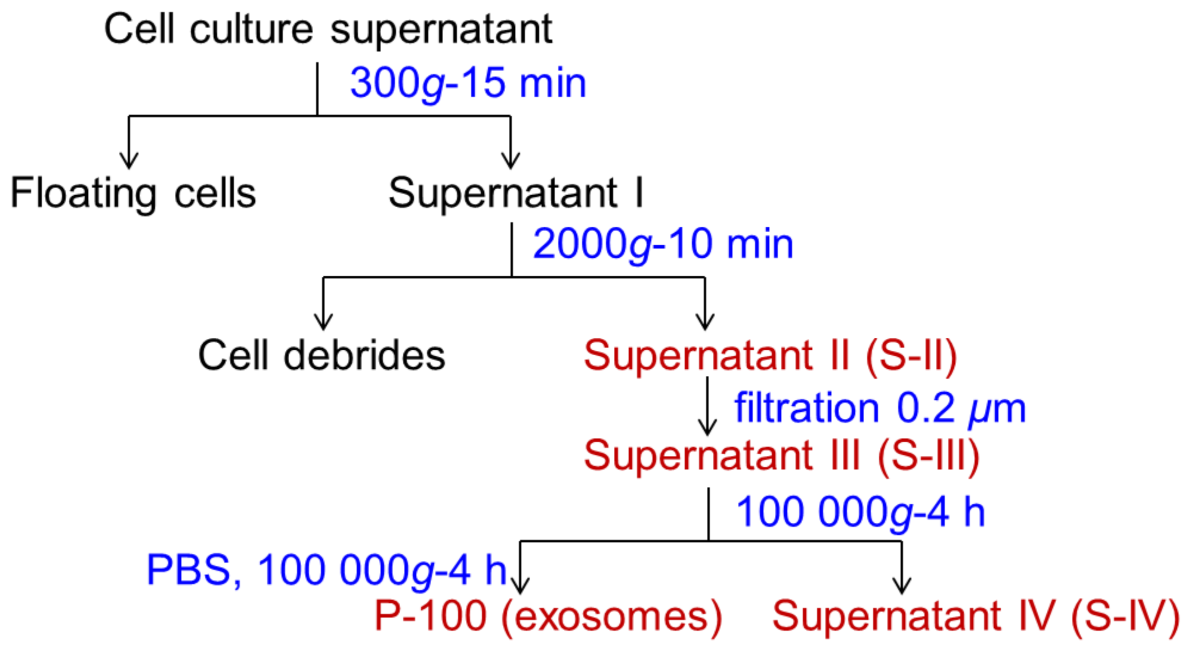

Figure S6. Negative correlations between VSTM2A mRNA and multiple Wnt target genes in the TCGA-COAD dataset.

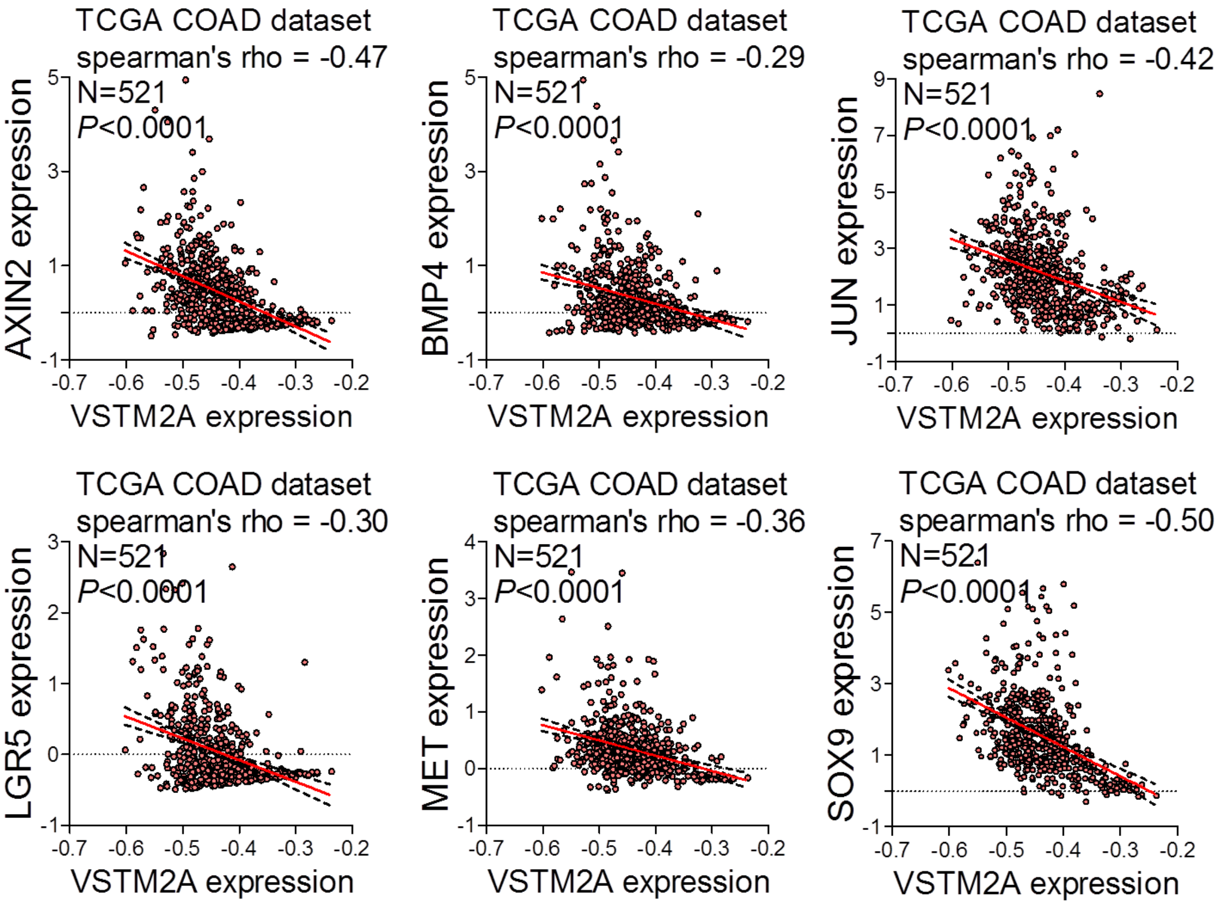

Figure S7. Ectopic expression of VSTM2A showed no effect on LRP6 mRNA level in RKO and 293T cells.

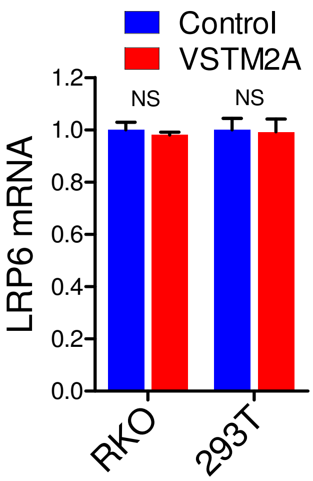

Figure S8. 293T cell surface proteins were biotinylated using a reversible biotinylation agent Sulfo-NHS-SS-Biotin. Following labelling, cells were stimulated with VSTM2A conditioned medium or control medium for the indicated times at 37°C. Cell was treated with sodium 2-mercaptoethanesulfonate to strip away any biotinylated proteins remaining on the cell surface. Whole cell lysis was precipitated with Dynabeads® MyOne™ Streptavidin C1 followed by immunoblot analysis with anti LRP6 antibody.

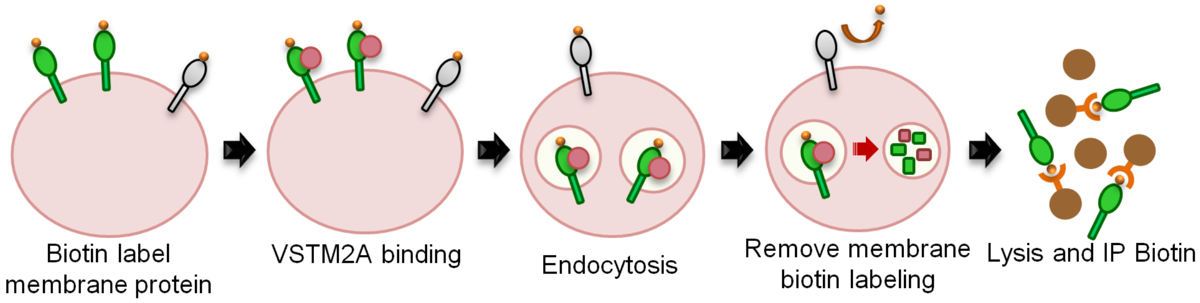

Figure S9. Silencing efficiency of siRNA targeting LRP6 was tested by QPCR and Western blot.

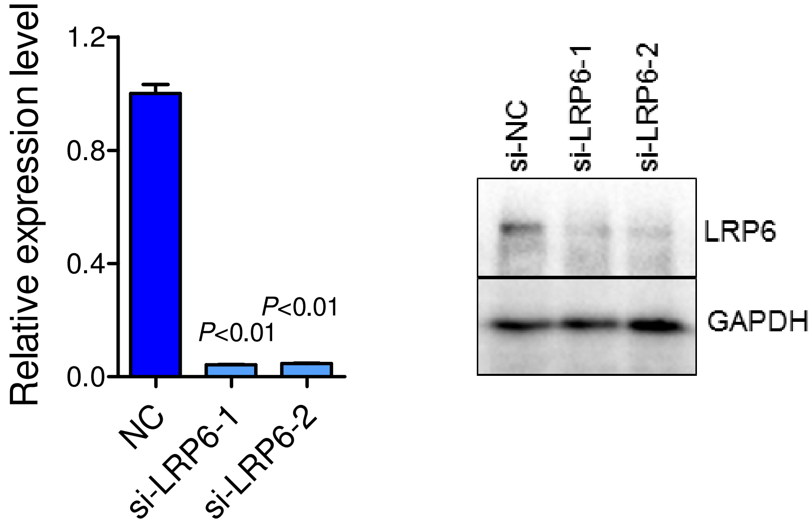

Figure S10. BGS analysis results of VSTM2A CpG islands 15-40 in seven CRC cell lines and four normal colon specimens are depicted. Dense methylation was observed in CRC cell lines, but not in normal colon tissue.

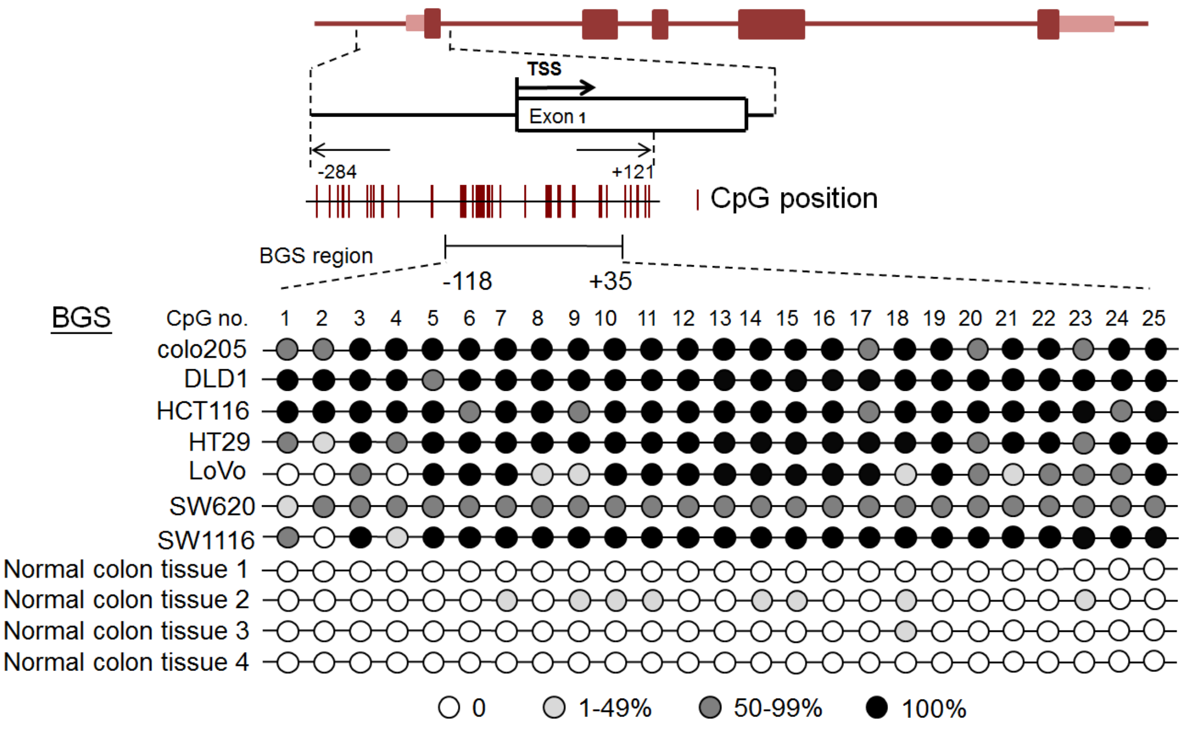

Figure S11. Effects of 5-Aza and TSA on LRP6 and Wnt downstream targets in CRC cell lines. (*, $P < 0.05$; ** $P < 0.01$; *** $P < 0.001$, NS, not significant)

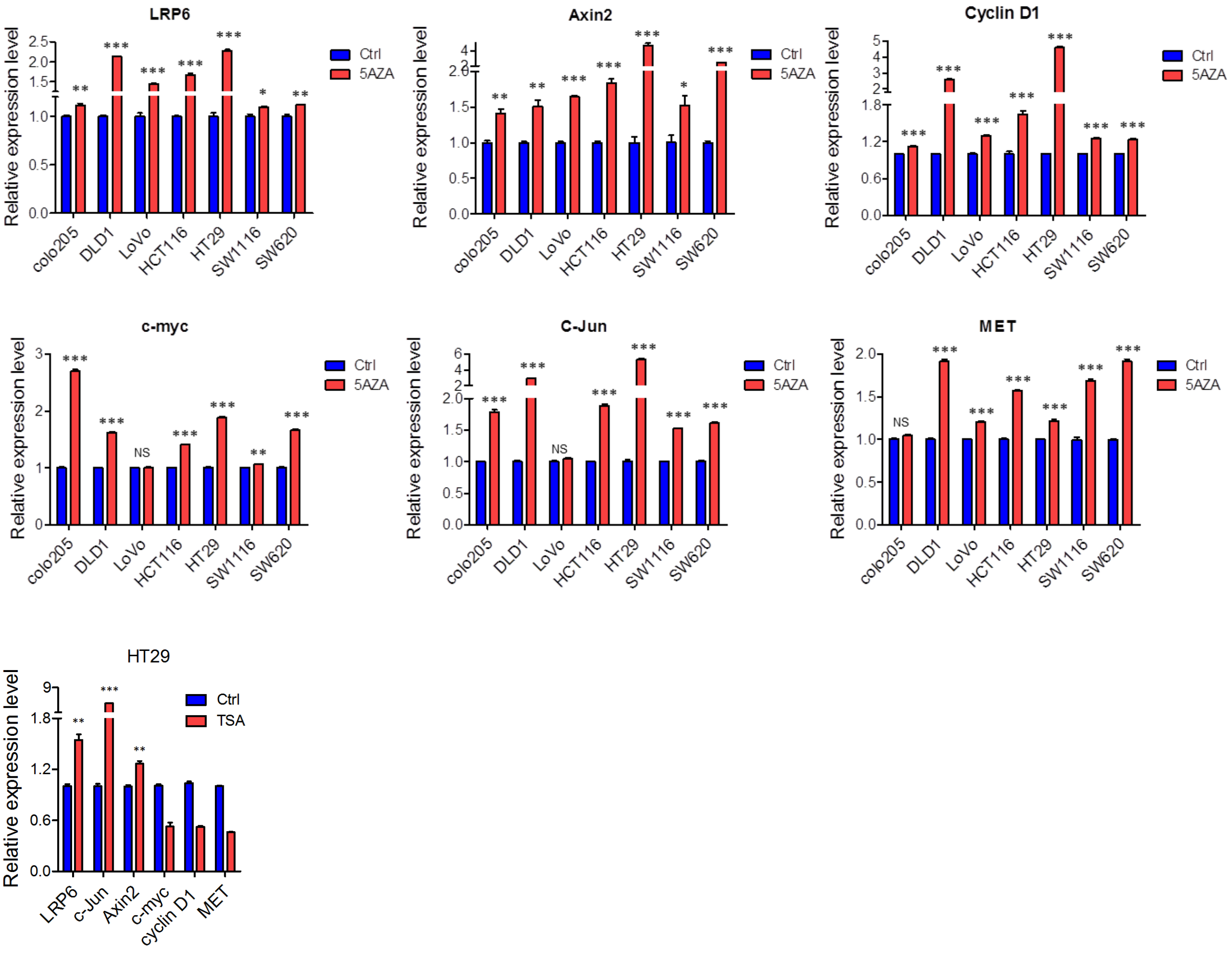

Figure S12. BGS analysis results of VSTM2A CpG islands 15-40 in 10 primary tumors and matched adjacent nontumor tissues from CRC patients are depicted.

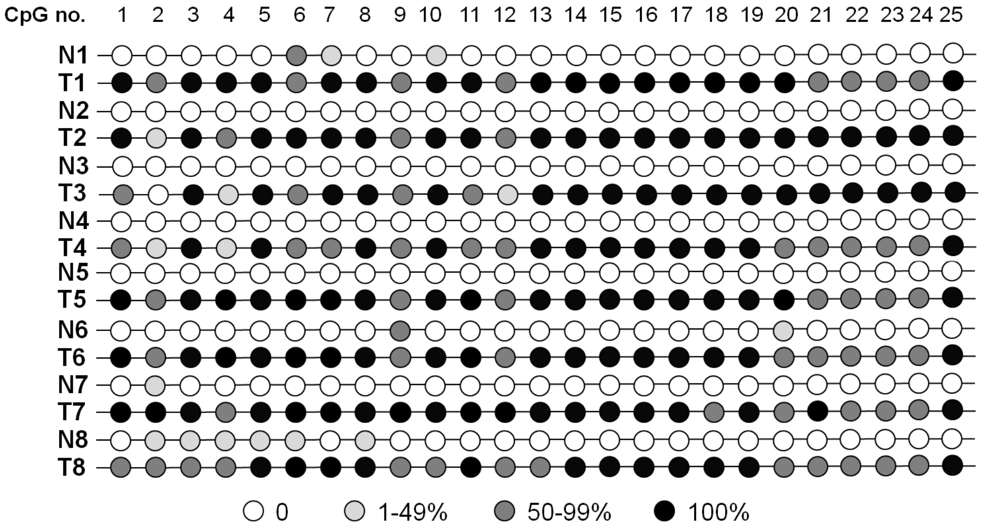

Figure S13. Six CpG probes located at VSTM2A promoter region was increase in tumor tissue and negatively associate with VSTM2A mRNA.

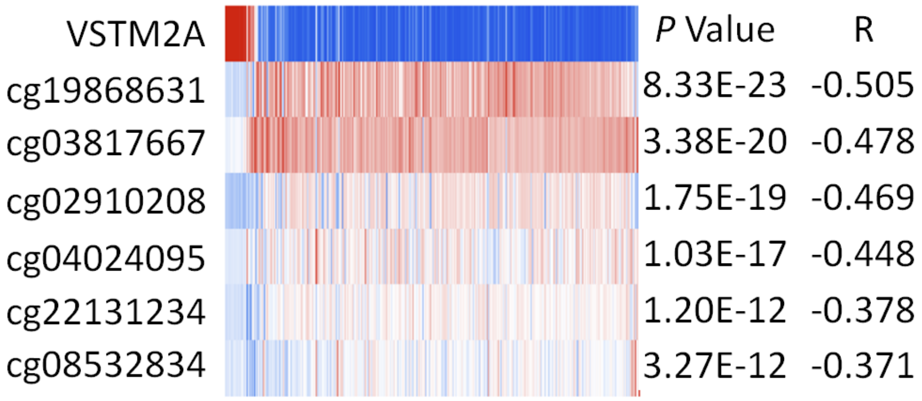

Figure S14. VSTM2A overexpression in HCT116 and DLD1 was detected by western blot.

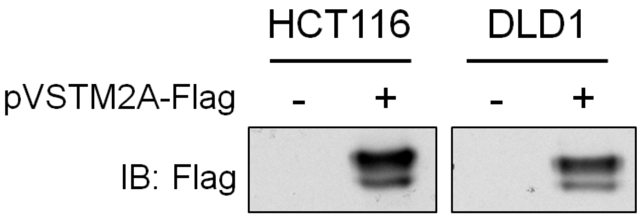

Supplementary Table

Table S1. DNA Sequences of primers

**Primer names**	**Sequence (5'->3')**
**PCR**	
VSTM2A Q-F	GGACCAAGATCAGCACAGTGAA
VSTM2A Q-R	TCATCCTTTTTCCTCACTTTGGA
VSTM2A isoform 1, 3-F	ACCTCCAGCCCTCAAGTGGTA
VSTM2A isoform 1-R	CCTTTCTGTGGAGCGTTTGTG
VSTM2A isoform 3-R	ACAAAGCCACAAACAAGAAGCA
VSTM2A isoform 2-F	TCTCCAATCCTTCCAGAAGTCAA
VSTM2A isoform 2-R	TCTGTGTGACGTTGGGAAGGT
VSTM2A isoform 4-F	ACAATCAGCAAAGAGCAAATCG
VSTM2A isoform 4-R	GCAGGCGCTCCTTGTGTAGT
β-catenin Q-F	TCCTGAGGAAGAGGATGTGGAT
β-catenin Q-R	CCTCTGAGCTCGAGTCATTGC
Cyclin D1 Q-F	CTGGAGGTCTGCGAGGAACA
Cyclin D1 Q-R	AGCTGCAGGCGGCTCTTT
c-myc Q-F	GAGCCCCTGGTGCTCCAT
c-myc Q-R	GCCTGCCTCTTTTCCACAGA
β-actin F	GTCTTCCCCTCCATCGTG
β-actin R	AGGGTGAGGATGCCTCTCTT
GAPDH-F	GCACCACCAACTGCTTAGCA
GAPDH-R	TCTTCTGGGTGGCAGTGATG
**BGS**	
VSTM2A-BGS-F	TGTTTTTTAGAAGGGAGATTTTAG
VSTM2A-BGS-R	TACCTAACTACCTACTTTCAACTACCTAAC

Abbreviations: PCR: polymerase chain reaction; BGS, bisulfite genomic sequencing.

Table S2. A list shows the antibodies used.

	Company	Catalog No.	Dilution	note
VSTM2A	Sigma-Aldrich	HPA024134	1:1,000	WB
VSTM2A	Sigma-Aldrich	HPA024134	1:50	IHC
β-catenin	Cell Signaling	#9562	1:4,000	WB
Active β-Catenin	millipore	05-665	1:1,000	WB
LRP6	Cell Signaling	#2526	1:1,000	WB
p-LRP6 (Ser1490)	Cell Signaling	#2568	1:1,000	WB
PCNA	Cell Signaling	#2586	1:1,000	WB
p27	Cell Signaling	#2552	1:1,000	WB
p21	Santa cruz	sc-6246	1:1,000	WB
Cyclin-D1	Santa cruz	sc-246	1:500	WB
c-myc	Santa cruz	sc-40	1:1,000	WB
Cleaved caspase-3	Cell Signaling	#9661	1:200	WB
Cleaved caspase-8	Cell Signaling	#9496	1:500	WB
Cleaved PARP	Cell Signaling	#9541	1:500	WB
Flag-tag	Sigma-Aldrich	F1804	1:1,000	WB
HA-tag	Santa cruz	sc-7392	1:1,000	WB
GAPDH	Santa cruz	sc-25778	1:1,000	WB
Flag-tag	Sigma-Aldrich	F7425	1:5000	IF
Alexa Fluor® 594 Goat Anti-Rabbit IgG (H+L)	Thermo Fisher Scientific	A-11037	1:1,000	IF
Alexa Fluor® 488 Goat Anti-mouse IgG (H+L)	Thermo Fisher Scientific	A-11001	1:1,000	IF

Abbreviations: IF, immunofluorescence; IHC, Immunohistochemistry; WB, western blotting;.

Supplementary Table S3. Peptide of LRP6 identified by LC-MS/MS

Confidence	High	High	High	High	High	High
PSM Ambiguity	Unambiguous	Unambiguous	Unambiguous	Unambiguous	Unambiguous	Unambiguous
Sequence	QLYWIDSR	NLYWTDTGTDR	ELNLQEYR	IESSDLSGANR	NLYWTDTGTDR	ELNLQEYR
Annotated Sequence	[K].QLYWIDSR.[Q]	[R].NLYWTDTGTDR.[	[K].ELNLQEYR.[Q]	[R].IESSDLSGANR.[I]	[R].NLYWTDTGTDR.[	[K].ELNLQEYR.[Q]
Modifications						
Qvality PEP	0.0155085	0.000579723	0.00773777	0.000375503	0.000105046	0.026661
Qvality q-value	0.00209298	0.000390623	0.000663183	0	0.00115319	0.00200678
SVM_Score	0.212	0.824	0.292	0.985	1.019	0.165
# Protein Groups	1	1	1	1	1	1
# Proteins	6	8	7	6	8	7
# PSMs	1	1	1	1	1	1
Master Protein Accessions	O75581	O75581	O75581	O75581	O75581	O75581
Master Protein Descriptions	Low-density lipoprotein receptor-related protein 6 OS=Homo sapiens OX=9606 GN=LRP6 PE=1 SV=2					
Protein Accessions	Q59EX8; B3KQG5; H0YGW5; B7ZLD0; O75581; F5H7J9					
Positions in Master Proteins	O75581 [978-985]	O75581 [416-426]	O75581 [1196-1203]	O75581 [1126-1136]	O75581 [416-426]	O75581 [1196-1203]
# Missed Cleavages	0	0	0	0	0	0
Theo. MH+ [Da]	1080.54727	1341.60697	1064.5371	1148.55421	1341.60697	1064.5371
Sequence Length	8	11	8	11	11	8
# Razor Quan Results	0	0	0	0	0	0
Abundances (Scaled): F1: Sample	100	100		100	100	100
Abundances (Normalized): F1: Sample	168001.7813	207064.5		1391284.875	323562.0625	531327.3125
Abundance: F1: Sample	168001.7813	207064.5		1391284.875	323562.0625	531327.3125
Abundances Count: F1: Sample	1	1		1	1	1
Quan Info			NoQuanValues			
Found in Sample: [S1] F1: Sample	High	High	High	High	High	High
Found in Sample Group: [S1] F1: Sample	High	High	High	High	High	High
Confidence (by Search Engine): Sequest HT	High	High	High	High	High	High
Charge (by Search Engine): Sequest HT	2	2	2	2	2	2
DeltaScore (by Search Engine): Sequest HT	0.4857	0.6278	0.188	0.5969	0.4488	0.0638
DeltaCn (by Search Engine): Sequest HT	0	0	0	0	0	0
Rank (by Search Engine): Sequest HT	1	1	1	1	1	1
Search Engine Rank (by Search Engine): Sequest HT	1	1	1	1	1	1
Concatenated Rank (by Search Engine): Sequest HT	1	1	1	1	1	1
m/z [Da] (by Search Engine): Sequest HT	540.77802	671.30481	532.77429	574.78217	671.30505	532.77533
DeltaM [ppm] (by Search Engine): Sequest HT	1.37	-3.45	3.95	2.48	-3.09	5.9
Deltam/z [Da] (by Search Engine): Sequest HT	0.00074	-0.00231	0.0021	0.00142	-0.00207	0.00314
RT [min] (by Search Engine): Sequest HT	27.5487	23.1457	21.4898	12.5586	23.3075	21.8321
Percolator q-Value (by Search Engine): Sequest HT	0.002059	0.0003246	0.0009094	0	0.0009785	0.00169
Percolator PEP (by Search Engine): Sequest HT	0.01408	0.0006577	0.007484	0.0003987	9.25E-05	0.02478
Percolator SVMScore (by Search Engine): Sequest HT	0.212	0.824	0.292	0.985	1.019	0.165
XCorr (by Search Engine): Sequest HT	1.05	1.8	1.17	1.96	1.27	0.94
Top Apex RT [min]	27.53	23.14		12.66	23.42	21.66

Supplementary Tables

Table S4. **Correlation of VSTM2A expression with clinicopathologic parameters in 158 CRC patients.**

**Variable**	**VSTM2A expression**				
	**Low (N=80)**	**%**	**High (N=78)**	**%**	***P*-Value**
**Sex**					
Female	36	45.0%	28	35.9%	0.26
Male	44	55.0%	50	64.1%	
**Age**					
≤70	44	55.0%	35	44.9%	0.27
>70	36	45.0%	43	55.1%	
**Location**					
Colon	35	43.8%	30	38.5%	0.52
Rectal	45	56.2%	48	61.5%	
**Differentiation**					
Well/Moderate	76	95.0%	75	96.2%	1.00
Poor	4	5.0%	3	3.8%	
**Stage**					
I	8	10.0%	9	11.5%	0.11
II	29	36.2%	37	47.4%	
III	28	35.0%	27	34.6%	
IV	15	18.8%	5	6.4%	

Table S5. Univariate and multivariate Cox regression proportional hazards analysis of prognostic factors for overall survival in TCGA cohort (N = 395).

**Variable**	**Univariate analysis**		**Multivariate analysis**	
	**RR (95% CI)**	***P*-value**	**RR (95% CI)**	***P*-value**
Sex				
Female	0.60 (0.37-0.98)	0.040	0.82 (0.49-1.38)	0.471
Male	1.00		1.00	
Age				
≤60	0.53 (0.30-0.92)	0.025	0.38 (0.20-0.73)	0.004
>60	1.00		1.00	
Primary cancer location				
Colon	1.62 (0.83-3.16)	0.160	1.77 (0.86-3.63)	0.120
Rectum	1.00		1.00	
TNM stage				
I	0.14 (0.04-0.47)	0.002	0.13 (0.03-0.39)	0.001
II	0.27 (0.14-0.51)	<0.001	0.20 (0.10-0.41)	<0.001
III	0.38 (0.20-0.72)	0.003	0.34 (0.18-0.65)	0.001
IV	1.00		1.00	
***VSTM2A* methylation**				
**High methylation**	**2.03 (1.27-3.26)**	**0.003**	**1.67 (1.01-2.73)**	**0.044**
**Low methylation**	**1.00**		**1.00**	
